# Chiral Graphene Hybrid Materials: Structures, Properties, and Chiral Applications

**DOI:** 10.1002/advs.202003681

**Published:** 2021-02-12

**Authors:** Biao Zhao, Shenghua Yang, Jianping Deng, Kai Pan

**Affiliations:** ^1^ State Key Laboratory of Chemical Resource Engineering Beijing University of Chemical Technology Beijing 100029 China; ^2^ College of Materials Science and Engineering Beijing University of Chemical Technology Beijing 100029 China

**Keywords:** chiral applications, chirality, graphene, hybrid materials

## Abstract

Chirality has become an important research subject. The research areas associated with chirality are under substantial development. Meanwhile, graphene is a rapidly growing star material and has hard‐wired into diverse disciplines. Rational combination of graphene and chirality undoubtedly creates unprecedented functional materials and may also lead to great findings. This hypothesis has been clearly justified by the sizable number of studies. Unfortunately, there has not been any previous review paper summarizing the scattered studies and advancements on this topic so far. This overview paper attempts to review the progress made in chiral materials developed from graphene and their derivatives, with the hope of providing a systemic knowledge about the construction of chiral graphenes and chiral applications thereof. Recently emerging directions, existing challenges, and future perspectives are also presented. It is hoped this paper will arouse more interest and promote further faster progress in these significant research areas.

## Graphical abstract

Chiral graphene hybrid materials have emerged as a rising star in multidisciplinary fields with significant potentials. This review paper systematically summarizes the progress made in chiral graphene hybrid materials, with the hope to provide a systemic knowledge of the construction of chiral graphenes and their chiral applications. Recently emerging directions, existing challenges, and future perspectives are also presented.

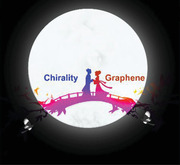

## 1 Introduction

Chirality is an essentially fundamental attribute of nature. Its implications have been well recognized in both fundamental research and industry, in particular from the disclosure of the course for the “thalidomide” tragedy.^[^ ^1^ ^]^ From then on, rapidly growing interest has been devoted to studies dealing with chirality, among which the development of chiral drugs and chiral agrochemicals are the representative.^[^ ^2^, ^3^, ^4^, ^5^ ^]^ Chirality has been infiltrated into various research areas including chemistry, material, pharmaceutical, bioengineering, agriculture, environment, etc. Undoubtedly, chirality has become one of the foremost “elements” indispensable for human being to keep healthy and living normal life.^[^ ^6^ ^]^ For the development of science and technologies, design and exploration of powerful materials are one of the necessary prerequisite research themes. This is also true for chirality‐associated academic research and industry applications. Numerous chiral materials have been created and are still under active exploitation for uses in chiral separation,^[^ ^7^ ^]^ asymmetric catalysis,^[^ ^8^, ^9^, ^10^ ^]^ chiral recognition/sensing,^[^ ^11^, ^12^ ^]^ and even chiral illuminant materials.^[^ ^13^, ^14^ ^]^ Accordingly, combining chirality with advanced materials will surely lead to new materials with promising potential uses. This has been well exemplified by the successful integration of graphene family materials and chirality.

In the last decades, 2‐D materials began to gather wide interest due to their unique structures and huge potential applications. As a typical 2D material, graphene has attracted large attention since it was first reported in 2004.^[^ ^15^ ^]^ Graphene and its analogues, primarily graphene oxide (GO), and reduced graphene oxide (RGO), currently still draw rapidly increasing attention of scientists from multidisciplines worldwide.^[^ ^16^, ^17^, ^18^ ^]^ Graphene has distinctive nanostructure and a variety of superior thermal, mechanical, and electrical properties. The fascinating structure and properties make graphene‐family materials hold large promise for potential applications in many areas including catalysis,^[^ ^19^, ^20^, ^21^ ^]^ sensors,^[^ ^22^, ^23^ ^]^ electronic/photonic devices,^[^ ^24^, ^25^, ^26^ ^]^ inorganic/metallic nanocomposites,^[^ ^27^, ^28^ ^]^ supercapacitors,^[^ ^29^ ^]^ energy storage,^[^ ^30^ ^]^ batteries,^[^ ^31^ ^]^ and biological and nanomedicine applications.^[^ ^32^, ^33^, ^34^ ^]^ Excellent review papers provide comprehensive overviews on graphene family materials from fundamental, structure, properties and applications.^[^ ^19^, ^20^, ^21^, ^22^, ^23^, ^24^, ^25^, ^26^, ^27^, ^28^, ^29^, ^30^ ^]^ Aside from the special 2D nanostructure features and the corresponding thermal and conducting superiority, graphene also has high surface area (calculated value, 2630 m^2^ g^−1^).^[^ ^35^ ^]^ This additional attribute enables graphene to be an ideal candidate for developing materials toward separation/adsorption and as carriers for catalysts.^[^ ^36^, ^37^ ^]^ GO retains the basic properties of graphene with sp^2^‐hybridized carbons on the aromatic network, but meanwhile contains sp^3^‐hybridized carbons bearing hydroxyl, carboxyl, and epoxide functional groups on the top and bottom surface and edges. The presence of the rich oxygen‐containing groups endows GO sheets with significant capability of further functionalization via covalent bonds and noncovalent interactions.^[^ ^16^, ^17^, ^18^ ^]^ Exploiting functionalized GO materials has become one of the most hot research topics especially in nanomedicine and bioengineering areas.^[^ ^32^, ^33^ ^]^ The efforts have provided innumerable advanced functional materials, nearly having been hard‐wired into all science and technology fields. Among the numerous research directions concerning graphene‐derived functional materials, there is one intriguing research topic dealing with the connection of graphene with chirality, which is still filled up with various mystery even today.

Chirality, as an intriguing natural phenomenon extensively existing in nearly all scaled structures, has been exploited in multitude areas, in particular chemistry, biochemistry, medicine and pharmacology. Graphene, as a rapidly rising star and typical 2D material, has exhibited huge potentials in diverse fields. If graphene family materials and chirality are rationally combined in one single entity, the resulting chiral materials are anticipated to demonstrate series of superior properties and find a number of uses. This consideration has been justified by the myriad reports in literature. So far graphene‐based chiral materials have advanced substantially and are still in rapid progress, owing to judiciously combining the advantages of graphene and those derived from chiral structures. Even though graphene can be awarded with structural chirality through special processes like twisted stacking and buckling,^[^ ^38^, ^39^, ^40^ ^]^ chiral postfunctionalization using other chiral substances (e.g., chiral small molecules and chiral macromolecules) constitutes the most popular way for developing chiral graphene materials. This is the case especially with GO. GO contains abundant oxygen‐containing active groups including hydroxyl, carboxyl, and epoxide groups. These reactive groups provide powerful and versatile “handles” to covalently graft achiral and chiral moieties onto graphene sheets.^[^ ^16^, ^17^, ^18^, ^20^, ^29^, ^31^ ^]^ In addition, noncovalent interactions also can be taken to attach chiral moieties onto graphene sheets due to the 2D architecture and the oxygen‐containing functional moieties.^[^ ^41^, ^42^, ^43^ ^]^ The noncovalent interactions may be hydrophobic effect, *π*–*π* stacking interaction, electrostatic effect, and so on. Moreover, the specific 2D architecture of graphene sheets facilitates them to load a considerable amount of external substances, implementing highly efficient utilization of surface area since both sides and edges of each graphene sheet are all easily available.

**Scheme 1:**
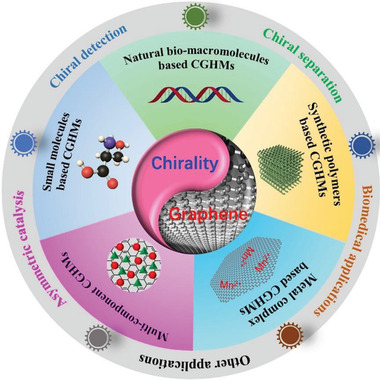
Schematic of chiral graphene hybrid materials (CGHMs) and their chiral applications.

Pristine graphene sheets tend to accumulate because of the hydrophobic skeleton, which is undesirable for graphene layers to retain a homogeneously dispersed state. Attachment of chiral components on graphene for chiral functionalization simultaneously helps to overcome this intractable trouble. Up to date, a great number of chiral sources have been taken to create graphene‐based chiral hybrid materials, from organic molecules (small organic molecules,^[^ ^44^, ^45^ ^]^ synthetic polymers,^[^ ^46^ ^]^ and natural macromolecules^[^ ^47^ ^]^) to much more completed chiral substances consisting of metal elements and chiral organic components (metal complexes).^[^ ^48^, ^49^ ^]^ Self‐assembly processes are also frequently used for constructing graphene‐containing functional chiral materials.^[^ ^50^, ^51^, ^52^ ^]^ Generally, these chiral hybrid materials were designed and explored with definitely intended applications, that is, as chiral catalysts,^[^ ^53^ ^]^ chiral separating media,^[^ ^54^ ^]^ chiral detectors (including chiral recognition materials and sensors),^[^ ^55^ ^]^ and in biomedical area.^[^ ^56^ ^]^ Striking advancements have been made; however, so far there has been no such a review paper to systemically summarize the scattered studies, most likely because of the multiple disciplines involved in a topic under discussion. To demonstrate the significant advancements and in particular to promote future progress, this comprehensive overview paper is prepared accordingly. It summarizes the state‐of‐the art studies focused on chiral graphene hybrid materials (CGHMs), as illustrated in **Scheme** **1**. The organization of the paper is as follows.

The first part is Introduction. The second part (Section 2) mainly introduces the diverse chiral hybrid materials constructed by graphene (GO and RGO), which are classified according to the types of chiral components (chiral sources, grouped into: chiral small molecules, natural biomacromolecules, synthetic polymers, and metal complexes) constituting the chiral hybrids, followed by a separation sub‐section dealing with multiple component‐constructed chiral graphenes. In Section 3, the contents are arranged according to chiral applications of the resulting chiral hybrids, falling into primarily five groups: as chiral catalysts catalyzing asymmetric reactions; as chiral detectors for enantioselective sensing and recognition; as chiral selectors for chiral separation (chiral stationary phases for chromatography, chiral membranes, enantioselective crystallization, and enantioselective adsorption); biological applications and others. Section 4 presents some newly emerging graphene based chiral architectures. In Section 5, the perceived challenges are discussed, followed by future perspectives correlated with the research field. Herein, we point out that, for the purpose of clarity, graphene and the derivatives (predominantly graphene oxide, GO, and reduced GO, RGO/rGO) in this overview paper are totally unified as graphene, unless it needs a clear indication. RGO and rGO are simultaneously used in this review as both of them appeared in the figures cited from the literature. In addition, all the GO in the cited references were prepared by Hummers’ method with minor modifications.^[^ ^57^ ^]^ This will not be repeated in the following contents.

## 2 Construction of Chiral Graphene Hybrid Materials

### 2.1 Small Organic Molecules as Chiral Source

#### 2.1.1 Amino Acids

**Figure 1:**
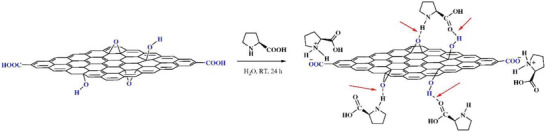
Illustration of preparing l‐proline/GO hybrid. l‐proline immobilized on GO sheets via hydrogen bonds. The red arrows demonstrate hydrogen bonds. Reproduced with permission.^[^ ^41^ ^]^ Copyright 2012, Elsevier.

The amino acids used to combine with graphene are summarized in **Table** **1**, which also presents the chiral application of the resulting chiral hybrid products. Amino acids play crucial roles in biological organisms and are commonly found in foods, feeds, body fluids and tissues. Inarguably, they constitute one of the most predominant and important chiral compounds in nature. It had been assumed for a long time that l‐amino acids were solely found in nature and had biological and pharmaceutical activity, while D‐isomers were laboratory artifacts and possessed therapeutic ineffectiveness or even led to negative effects on living organisms. However, intense investigations have demonstrated that d‐amino acids are also widespread in mammals and humans.^[^ ^63^ ^]^ Now it has been clearly apparent that the total amount and enantiomeric excess (e.e.) of amino acids play vital roles in human being health. If error occurs to these conditions, neurological disorders may appear like Parkinson's, Huntington's and Alzheimer's disease.^[^ ^64^ ^]^ Typical amino acids have at least one carboxyl group and one amino group. The reactive functional groups facilitate amino acids to readily undergo chemical reaction with GO. In addition, the polar moieties also render amino acids with capability of forming hydrogen bonds with the functional groups on GO sheets. Typically, Yin group used proline to chirally functionalize GO, with the aim to develop a hybrid chiral catalyst.^[^ ^41^ ^]^ A variety of materials have been taken as supports to immobilize proline and its derivatives. The supports can be polymers,^[^ ^65^, ^66^ ^]^ inorganic materials like silica^[^ ^67^ ^]^ and magnetic nanoparticles,^[^ ^68^ ^]^ cyclodextrins,^[^ ^69^ ^]^ and ionic liquids.^[^ ^70^ ^]^ The layered structure and desirable properties of graphene enable it to be a new alternative for acting as supporting material for immobilizing proline. Characterizations demonstrate that l‐proline could be efficiently loaded on the two sides and edges of GO layers through hydrogen bonding and/or ionic interaction. The noncovalent interactions between proline with GO is illustratively shown in **Figure** **1**.^[^ ^41^ ^]^ The resulting chiral hybrid was subsequently used as chiral catalyst for catalyzing direct aldol reaction. Considering the highly similar molecular structures of chiral amino acids, the work from Yin can serve as a versatile platform for using other amino acids for chiral functionalization of GO. Moreover, diverse amino acids are anticipated to give rise to chiral hybrids with individual properties and applications.

**Table 1:** Overview of amino acids used for chiral functionalization of graphene and applications of the chiral hybrid materials

Chiral amino acid	Graphene (GO, RGO)	Chiral applications^a)^	Ref.
l‐Proline	GO	Chiral catalyst (aldol reaction)	^[^ ^41^, ^43^, ^53^ ^]^
l‐Proline	GO	Chiral catalyst (ketene forming reaction)	^[^ ^58^ ^]^
l‐Phenylalanine	GO	Chiral membrane for separation (phenylalanine, methionine, *N*‐acyl‐phenylalanine, *N*‐acyl‐methionine racemic enantiomers)	^[^ ^59^ ^]^
Boc‐l‐phenylalanine	Graphene	Electrochemical chiral sensing (*β*‐citronellol)	^[^ ^42^ ^]^
l‐Glutamic acid	GO	Chiral membrane for separation (3,4‐dihydroxy‐phenylalanine enantiomers)	^[^ ^45^ ^]^
l‐Glutamic acid	GO	Electrochemical chiral recognition (3,4‐dihydroxy‐phenylalanine enantiomers)	^[^ ^60^ ^]^
l‐Glutamic acid	GO	Chiral membrane for separation (3,4‐dihydroxy‐l‐phenylalanine enantiomers)	^[^ ^61^ ^]^
d‐ and l‐cysteine	GO	Electrochemical chiral recognition (tartrate enantiomers)	^[^ ^54^ ^]^
l‐Lysine	RGO	Electrochemical chiral discrimination (tryptophan enantiomers)	^[^ ^62^ ^]^

^a)^ In the brackets, the corresponding asymmetric reaction and chiral analytes for enantioselective detection, adsorption, release, or crystallization are presented.

Besides pristine amino acids, their derivatives alike also can be attached to graphene layers for diverse purposes. Kwak and co‐workers first chiral‐functionalized pyrene with Boc‐l‐phenylalanine and then combined the chiral product with graphene through noncovalent contacts.^[^ ^42^ ^]^ Herein pyrene moieties were selected to act as fluorophore. The as‐prepared chiral composite worked as chiral chemical sensors for enantioselective detection of natural acryclic monoterpenoid enantiomers, (*R*)‐(+)‐ and (*S*)‐(−)‐*β*‐citronellol. The chiral sensors show high sensitivity and selectivity. In view of the large number of amino acids and their potential chiral derivatives and the numerous fluorophores as well, innumerable chiral sensors and even other chiral materials and devices can be constructed based on similar building blocks, following the preparation strategy created in the study.^[^ ^42^ ^]^ Despite chiral detection has great potential to be applied in many areas, it currently still remains challenging in practice due to the substantial difficulty in discriminating enantiomers that possess the same chemical composition and nearly identical physical properties and differs only in steric conformation. The chemical sensors prepared through integrating a chiral moiety, a fluorophore, and graphene may provide straightforward and effective tools for chiral discrimination.

**Figure 2:**
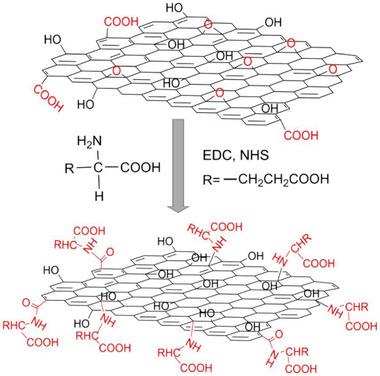
A typical illustration for the reactions between amino acid and functional groups on GO sheet. EDC, 1‐(3‐dimethyl‐aminopropyl)‐3‐ethylcarbodiimide methiodide; NHS, *N*‐hydroxysulfosuccinimide sodium salt. Reproduced with permission.^[^ ^45^ ^]^ Copyright 2016, Elsevier.

Apart from noncovalent approaches (hydrophobic effects, *π*–*π* stacking, electrostatic interactions, etc.) for attachment of amino acids onto graphene,^[^ ^41^, ^42^, ^43^ ^]^ covalent bonds provide more choices and versatile approaches for the purpose of chiral functionalization of graphene. The abundant variety and number of reactive functional groups contained on GO sheets make covalent bonding between GO and amino acids highly feasible. Compared with noncovalent interactions, GO layers and amino acid moieties covalently bond together show higher stability.^[^ ^53^, ^58^ ^]^ Making a full use of the carboxyl and epoxide groups on GO to respectively undergo amidation reaction (carboxyl groups) and epoxide ring‐opening reaction followed by amination reaction (epoxide groups) with the amino functionality on amino acids is a simple and popular approach to chiralizing GO.^[^ ^45^, ^71^ ^]^ A typical illustration for preparing chiral hybrid materials is presented in **Figure** **2**, by which glutamic acid was efficiently attached on GO sheets by means of covalent bonds.^[^ ^45^ ^]^ Zhang et al. established another process for grafting amino acid onto GO.^[^ ^58^ ^]^ Making use of the hydroxyl and epoxide groups on GO sheets, carboxyl groups are introduced on GO first and then undergo esterification reaction with hydroxyl group in 4‐hydroxyl‐l‐proline. Taking the method, l‐proline was covalently anchored on GO. The graphene/l‐proline constructed chiral hybrids have proved to be good heterogeneous chiral catalyst especially for catalyzing direct asymmetric aldol reaction, as to be discussed in Section 3.

Durmaz et al. took a different route to introduce proline onto GO, in which aminopropyltriethoxysilane (APTES) first reacted with GO, resulting in GO‐NH_2_.^[^ ^53^ ^]^ The product was further subjected to amidation reaction between the amino groups on GO and the carboxyl groups in proline. The chiral product catalyzed aldol reaction under mild conditions (yield up to 88%, e.e. up to 85%). In fact, apart from chiral proline, many studies have demonstrated that graphene is a robust catalyst support for immobilizing catalytically active organic molecules, the hybrid catalysts from which can catalyze a variety of reactions including oxidation reactions,^[^ ^72^ ^]^ Friedel–Crafts reactions,^[^ ^73^ ^]^ Aldol/Michael additions,^[^ ^74^, ^75^ ^]^ epoxide ring‐opening reactions,^[^ ^76^ ^]^ one‐pot reactions,^[^ ^77^ ^]^ multicomponent reactions,^[^ ^78^ ^]^ and even polymerizations.^[^ ^79^ ^]^ The powerful catalytic capability of the resulting hybrid catalysts is attributed to the advantages of graphene, including large surface area, strong adsorption capacity, and high chemical stability. Easy attachment of catalytic moieties on graphene, in particular GO sheets, is another driving force for the research area to keep active. Additionally, graphene and the derivatives have a flat sheet‐like structure and a single layer of carbon atoms, thus providing a large open surface area that is readily accessible to reactants with low mass transfer resistance. Much more hybrid catalysts of the type, including both chiral and achiral ones based on graphene, are under intense development at present.^[^ ^19^, ^20^ ^]^ Considering the milestone implications of catalysts in the development history of chemistry academia and chemical industry, the endeavors along the direction will as always maintain hot in the foreseeing future. The studies are expected to lead to far‐reaching impacts in both fundamental research and industrial events.

**Figure 3:**
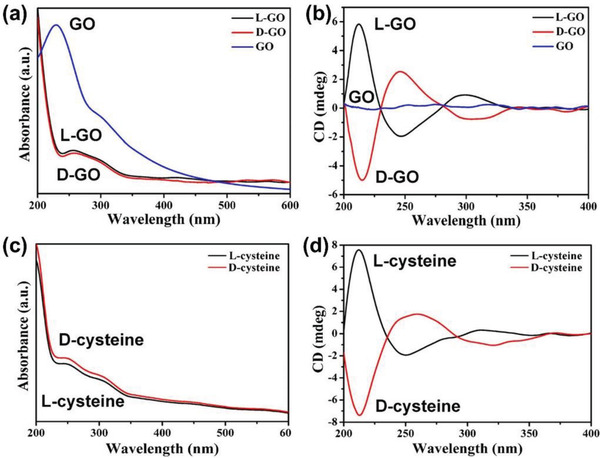
a) UV–vis absorption and b) CD spectra of l‐GO, d‐GO, and GO. c) UV–vis absorption and d) CD spectra of l‐ and d‐cysteine. l‐GO and d‐GO are GO combined with l‐ and d‐cystine, respectively. Reproduced with permission.^[^ ^54^ ^]^ Copyright 2019, Springer Nature.

Hydrothermal process can also allow covalent attachment of amino acids onto GO. Huang et al. reported a one‐step hydrothermal method that was used to graft cysteine on GO sheets.^[^ ^54^ ^]^ The chiralized GO was characterized by circular dichroism (CD) and UV–vis absorption spectroscopy to elucidate its optical activity. For recording the spectra, the chiral GO was dispersed in deionized water and a homogeneous aqueous dispersion was obtained. The recorded CD and UV–vis absorption spectra are presented in **Figure** **3**. The pure GO showed two UV–vis absorption peaks at 230 and 300 nm, in which the former one is ascribed to *π*–*π* conjugation of the C−C bonds in GO, whereas the latter reflects the n–*π* transition (Figure 3a). After chiralization with l‐ and d‐cystine (the corresponding product is called l‐GO and d‐GO, respectively), the two absorption peaks were overlapped by that of cystine. However, they showed similar absorption to those of l‐ and d‐cysteine (Figure 3a,c). CD spectra demonstrate that pure GO has no CD effect; l‐GO and d‐GO display apparent CD signals with high symmetry; their peaks are similar in magnitude to those derived from pure l‐ and d‐cysteine (Figure 3b,d). The spectra clearly show the success in chiralization of GO with chiral cysteine. Moreover, CD and UV–vis absorption spectra measured at different temperature and pH condition indicate the stability of the combination of cysteine with GO, due to covalent bonding occurring between them. The chiral GO was further used to modify glassy carbon electrode for recognition of tartrates. Synergistic effects were observed in the enantioselective recognition, in which GO increases the conductivity and cysteine provides a chiral environment. The versatile method is expected to be adaptable to other chiral molecules. Accordingly, the study opens an alternative avenue for preparing chiral GO, giving rise to a great number of chiral graphene materials.

More other processes have been exploited to covalently bond amino acids to GO. Mo et al. developed a three‐step route to attach l‐lysine on graphene: step‐1, preparation of lysine–copper–lysine (Lys–Cu–Lys) complex and attachment of it onto GO sheets through the terminal amino groups in the complex; step‐2, reduction of GO with NaBH_4_; and step‐3, elimination of the copper ions to form the chiral nanocomposite hybrid.^[^ ^80^ ^]^ The built method is potentially usable for functionalization of other substrate materials aside from GO. Meanwhile, it is also potentially applicable to other amino acids and even other chiral molecules for attaching them on diverse substrates. The idea of introduction of metal complex may be taken to develop metal complex catalysts through selecting appropriate metal elements and suited chiral ligands. The work thus deserves much more attention from multiple aspects (chemistry, materials, etc.), and such endeavors may lead to new chiral materials and even more chiral phenomena that cannot be observed in usual chiral material systems. The as‐obtained chiral hybrids may be subsequently exploited as chiral nanocomposite sensors, biological imaging, supercapacitors, among many others.

#### 2.1.2 Cyclodextrins

Cyclodextrins, frequently used in the form of *α*‐, *β*‐, and *γ*‐cyclodextrins (*α*‐CD, *β*‐CD, and *γ*‐CD, respectively^[^ ^81^ ^]^), constitute another prevalent category of chiral sources for chiral functionalization of both graphene and GO. Nonetheless, obviously different from amino acids, cyclodextrins are incorporated with graphene and the derivatives much more through noncovalent interactions^[^ ^82^, ^83^, ^84^, ^85^, ^86^ ^]^ rather than covalent bonding,^[^ ^87^, ^88^, ^89^ ^]^ probably because of the relatively more difficulties in accomplishing reactions between cyclodextrins and graphene. Another reason may lie in the fact that substitution of the initial —OH groups on cyclodextrins may exert influence on their enantioselectivity and the ability to form inclusion complex with guest compounds.

No matter how cyclodextrin units are attached onto graphene, the unique molecular architecture of cyclodextrins (hydrophobic cavity and hydrophilic exterior) together with chiral nature makes them quite interesting for developing chiral functional materials. Enantioselective effects come into play when a pair of enantiomers form inclusion complex with cyclodextrin units, in which only the enantiomer well matched with the cyclodextrin cavity in terms of stereostructure can efficiently form inclusion complex. In the course of forming inclusion complex, the chiral hydroxyl groups sited in the rim of the cyclodextrin holes serve as stereoselectors. Furthermore, cyclodextrins can encapsulate hydrophobic guest molecules and modify the latter ones’ water‐solubility. As a consequence, cyclodextrins have found a lot of practical applications in diverse industries like drug delivery and biomedical engineering,^[^ ^90^, ^91^ ^]^ synthetic chemistry,^[^ ^92^ ^]^ adsorption and separation,^[^ ^93^, ^94^ ^]^ food chemistry,^[^ ^95^ ^]^ and chiral recognition.^[^ ^96^ ^]^ Owing to the relatively more studies have been devoted to constructing cyclodextrin/graphene chiral material systems, the studies will be classified into two subgroups for a convenient discussion. The classification is performed according to whether cyclodextrins and graphene sheets are combined via noncovalent interactions or covalent interactions. The relevant studies are summarized in **Table** **2**.

**Table 2:** Overview of cyclodextrins used for chiral functionalization of graphene and applications of the chiral composite materials

Cyclodextrin	Graphene (GO, RGO)	Applications^a)^	Ref.
*α*‐, *β*‐, *γ*‐CD	Graphene	Electrochemical chiral sensing (DOPA)	^[^ ^82^ ^]^
*α*‐, *β*‐, *γ*‐CD	RGO	Electrochemical chiral differentiation (tartaric acid)	^[^ ^88^ ^]^
*α*‐CD	RGO	Electrochemical chiral detection (methionine)	^[^ ^83^ ^]^
*β*‐CD	RGO	Electrochemical chiral discrimination (cystine)	^[^ ^84^ ^]^
*β*‐CD	RGO	Electrochemical chiral recognition (phenylalanine)	^[^ ^85^ ^]^
*β*‐CD	RGO	Electrochemical chiral recognition (tryptophan)	^[^ ^86^ ^]^
*β*‐CD	GO	Chiral adsorption (phenylalanine, tryptophan, leucine, histidine, asparagine)	^[^ ^87^ ^]^
*β*‐CD	GO	Chiral sensing, chiral imaging	^[^ ^89^ ^]^
*β*‐CD	RGO	Electrochemical chiral recognition (Moxifloxacin hydrochloride)	^[^ ^97^ ^]^
*β*‐CD	RGO	Electrochemical chiral recognition (carboxylic acid)	^[^ ^98^ ^]^
*β*‐CD	RGO	Electrochemical chiral sensing (clopidogrel)	^[^ ^99^ ^]^
*β*‐CD	RGO	Electrochemical chiral recognition (phenylalanine)	^[^ ^100^ ^]^
*β*‐CD	GO	Electrochemical chiral recognition (phenylalanine)	^[^ ^101^ ^]^
*β*‐CD	GO	Chiral stationary phase for separation (tryptophan)	^[^ ^102^ ^]^
Methyl *β*‐CD	GO	Chiral stationary phase for separation (Naproxen, Warfarin, Pranoprofen)	^[^ ^103^ ^]^
*β*‐CD	GO	Chiral release (doxorubicin hydrochloride, DOX; epirubicin hydrochloride, EPI)	^[^ ^104^ ^]^
*β*‐CD	GO	Chiral recognition and separation (tryptophan)	^[^ ^105^ ^]^
*β*‐CD	GO	Electrochemical chiral recognition (phenylalanine)	^[^ ^106^ ^]^
Hydroxypropyl *β*‐CD	GO	Electrochemical chiral recognition (tryptophan)	^[^ ^107^ ^]^
*γ*‐CD	RGO	Electrochemical chiral sensing (tryptophan)	^[^ ^108^ ^]^

^a)^ In the brackets, the corresponding chiral analytes for chiral applications are presented.

##### Noncovalent Interactions

**Figure 4:**
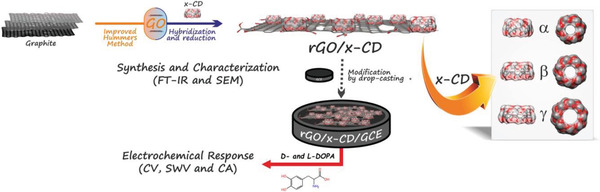
A schematic illustration for the preparation of rGO/x‐CD (*α*‐,*β*‐, and *γ*‐CDs) chiral hybrid and modification GCE with the prepared hybrid for sensing DOPA enantiomers. Reproduced with permission.^[^ ^82^ ^]^ Copyright 2017, Elsevier.

Graphene layer can be used as a scaffolding surface for anchoring cyclodextrin units, and the chiral product thereof can be utilized for diverse chiral applications. Massive efforts have been focused on this research topic. Currently, chiral hybrids of the type are still under active research. Zor and co‐workers developed a process to prepare chiral graphene modified electrode with the aim of discriminating chiral enantiomers.^[^ ^82^ ^]^ As presented in **Figure** **4**, the authors first prepared GO taking Hummers’ method with minor modification.^[^ ^57^ ^]^ Then GO was combined with cyclodextrins (x‐CD, x = *α*‐, *β*‐, or *γ*) to acquire GO/x‐CD hybrids, which were subsequently subjected to reduction, yielding RGO/x‐CD. In the next step, the RGO hybrids were taken to modify glassy carbon electrode (GCE), producing RGO/x‐CD/GCE that acted as chiral sensor for electrochemically recognizing 3,4‐dihydroxy‐phenylalanine (DOPA, the two enantiomers: l‐DOPA and d‐DOPA) enantiomers. Herein, RGO and cyclodextrin units were bonded via hydrogen bonds. According to electrochemical measurements, the RGO/*γ*‐CD nanohybrid was found to be able to enantioselectively recognize DOPA. The limit of detection value and sensitivity were respectively determined as 15.9 × 10^−6^ m and 0.2525 µA µm ^−1^ for d‐DOPA; for l‐DOPA, the values were 14.9 × 10^−6^ m and 0.6894 µA µm ^−1^ respectively. In addition, the authors measured the oxidation peak potentials of DOPA enantiomers by cyclodextrin/graphene nanohybrid through cyclic voltammetry (CV), square‐wave voltammetry (SWV), and chronoamperometry (CA) techniques. It demonstrated that only RGO/*γ*‐CD could trigger an obvious relative potential difference in redox peak. The experiment results clearly demonstrate the difference of *α*‐, *β*‐, and *γ*‐CDs in enantioselectivity is due to their ability to form inclusion complexes with guest analytes. The study lays a foundation for design and preparation of chiral hybrids potentially usable for other chiral sensing materials and chiral analytes.^[^ ^82^ ^]^ The developed graphene‐based chiral analysis system may be suitable for preparing chiral membranes for chiral separation, chiral films for enantioselective detection, and chiral absorbents for chiral capture. Moreover, the study also disclosed the difference among the three classes of cyclodextrins, i.e., *α*‐, *β*‐, and *γ*‐CDs. According to the size of the chiral analytes, a category of cyclodextrin is selected, whose cavity should have commensurate size with the analytes.

Molecular docking studies provide some inspiring information for elucidating the observed phenomena. Although the three sets of cyclodextrins (*α*‐, *β*‐, and *γ*‐CDs) are all large enough to allow DOPA molecules to enter and form inclusion complex, the guest molecules enter the cyclodextrins’ cavity in different position.^[^ ^82^ ^]^ More specifically, as for *α*‐ and *β*‐CDs, DOPA molecules penetrated into the cavities in vertical position, while in the case of *γ*‐CDs, DOPA molecules entered into the cavity in horizontal position. The difference in this entering position, i.e., DOPA molecules orientation, led to varied binding energy and binding constant values between cyclodextrin units and DOPA molecules. In addition, the chirality of *γ*‐cyclodextrin units contributed to the chiral recognition ability toward DOPA enantiomers. The work^[^ ^82^ ^]^ reflects the necessity of molecular simulation, which can provide some theoretical support for experimental results and even insights into experimental phenomena that are difficult to understand. This point will be stressed again in Section 5 in this review paper, due to the unique roles played by theoretical simulation. By the way, developing chiral detection material systems in which enantioselective excess (e.e.) values can be read directly during chiral differentiation is worthy to be devoted more efforts. This may become into a new research theme in developing new chiral sensors with more feasibility for practical uses.

Based on the chiral hybrids constructed by graphene and cyclodextrin, more other components can be introduced into this basic material combination for developing new advanced chiral materials. Fu et al. prepared chiral nanohybrids composed of three components, that is, *β*‐CD, platinum nanoparticles, and graphene, for modifying electrode. The resulting electrode could recognize tryptophan enantiomers.^[^ ^86^ ^]^ Other chiral hybrids exploited following the similar idea are RGO/*β*‐CD/CPE (carbon paste electrode) for electrochemically sensing moxifloxacin hydrochloride enantiomers;^[^ ^97^ ^]^ RGO/*β*‐CD/MB/GCE (MB, methylene blue) for electrochemically sensing chiral carboxylic acids;^[^ ^98^ ^]^ RGO/*β*‐CD for electrically and optically sensing tryptophan enantiomers;^[^ ^87^ ^]^ RGO/PNP/*β*‐CD/CPE (PNP, platinum nanoparticles) for electrochemically sensing chiral clopidogrels;^[^ ^99^ ^]^ RGO/PTCA/*β*‐CD (PTCA, 3,4,9,10‐perylene tetracarboxylic acid) for electrochemically recognizing chiral phenylalanine;^[^ ^100^ ^]^ RGO/ferrocene/*β*‐CD for electrochemically recognizing phenylalanine racemate.^[^ ^101^ ^]^ These studies evidently show the importance of electrochemical sensors for enantio‐differentiating chiral compounds, due to their merits particularly in easy and fast operation.

Besides the above chiral hybrids derived from graphene, cyclodextrins, and other components, which were designed and prepared for chiral sensing or recognition, there are other investigations for implementing diverse chiral applications. Qiu and co‐workers prepared GO/*β*‐CD chiral magnetic nanocomposites and used them as tunable stationary phase in open‐tubular capillary electrochromatography for the chiral separation of tryptophan enantiomers.^[^ ^102^ ^]^ In the aforementioned studies, GO and RGO were taken for constructing the chiral hybrids. In recent years, graphene quantum dots (GQDs) emerge as a new class of hot nanoarchitectures for developing advanced functional materials, which will be specifically introduced in a separate section later on.

##### Covalent Bonds

**Figure 5:**
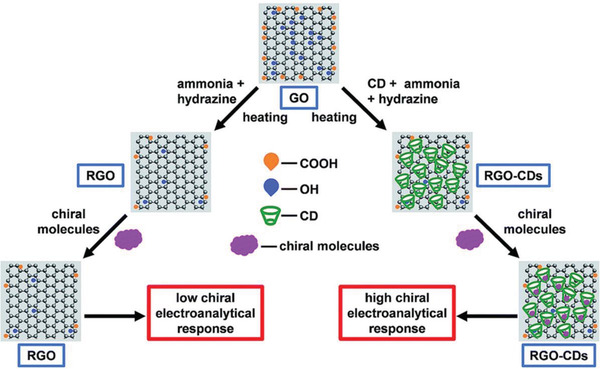
Illustration of the procedure for preparing RGO and RGO‐cyclodextrins for enantioselectively differentiating chiral molecules (tartaric acid). GO was reduced in the presence of (ammonia + hydrazine) to generate RGO. Pristine RGO served as a control sample. Reproduced with permission.^[^ ^88^ ^]^ Copyright 2018, Royal Society of Chemistry.

Noncovalent interactions provide straightforward processes for anchoring cyclodextrin units on graphene layers. But the stability of the chiral hybrids may be not satisfying enough for uses under certain conditions, especially at relatively higher temperature and in polar solvents. The rigorous conditions are not desirable for noncovalent bonds to retain stable. For further improving the stability, covalent bonds provide viable means for attachment of chiral molecules on graphene. Cheng and co‐workers built a convenient approach for covalently bonding cyclodextrins onto graphene.^[^ ^88^ ^]^ As shown in **Figure** **5**, GO was first prepared and then reduced with hydrazine hydrate to afford RGO, during which cyclodextrins (*α*,*β*, and *γ*‐CD) were added. The process finally led to RGO‐cyclodextrin chiral composites. Combined with glass carbon electrode technique, the chiral composites exhibited electrochemically enantioselective recognition ability toward tartaric acid racemate. Cyclic voltammetry tests show that among the three glass carbon electrodes modified respectively with *α*, *β*, or *γ*‐CD/RGO chiral composites, the one with *β*‐CD gave peak current with the best shape. This was explained by the more stable inclusion complex formed between *β*‐CD and the guest molecules. RGO without the attachment of cyclodextrin was taken as a control sample. It showed low chiral response, proving the roles of cyclodextrin units in the chiral discrimination. It is useful to point out that, theoretical simulation may help disclose the different performances observed in *α*, *β*, or *γ*‐CD units. Indeed, theoretical simulation has been intensely utilized to assist in understanding chiral processes and the relevant mechanisms, for example, in heterochiral to homochiral transition in crystallization,^[^ ^109^ ^]^ chiral recognition,^[^ ^110^ ^]^ and formation of enantioselective inclusion complex.^[^ ^111^ ^]^

**Figure 6:**
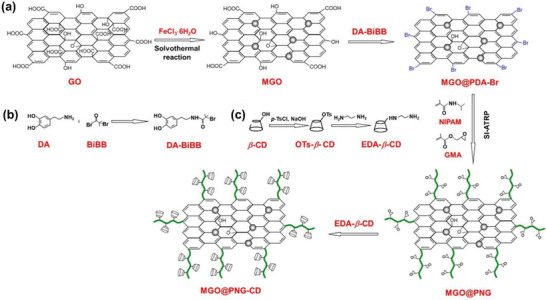
Major steps for preparing chiral hybrids consisting of GO, Fe_3_O_4_ nanoparticles, copolymer chains (PNG) from NIPAM and GMA, and *β*‐CDs. MGO, magnetic GO. SI‐ATRP, surface initiated ATRP. EDA‐*b*‐CD was grafted on MGO sheets through the reaction between —NH_2_ groups on EDA‐CD and the epoxide groups on GMA units in PNG. Reproduced with permission.^[^ ^105^ ^]^ Copyright 2018, American Chemical Society.

To enhance the amount of cyclodextrins attached on GO sheets and simultaneously to prepare smart and pH‐sensitive chiral hybrids, Cheng and co‐workers created a distinctive route for grafting cyclodextrin units on GO, as presented in **Figure** **6**.^[^ ^105^ ^]^ The produced chiral hybrids were composed of GO, Fe_3_O_4_ nanoparticles, poly(NIPAM‐*co*‐GMA) and *β*‐CD. The preparation of the target chiral hybrids can be divided into four major steps: 1) deposition of Fe_3_O_4_ nanoparticles on GO to fabricate magnetic GO (MGO); 2) grafting active sites for subsequently initiating free radical polymerization on MGO, thereby forming MGO@PDA‐Br; 3) surface‐initiating atom transfer radical polymerization (ATRP) of NIPAM and GMA monomers to generate MGO@PNG (poly(NIPAM‐*co*‐GMA)); 4) grafting *β*‐CD units along the copolymer chains via epoxide ring‐opening reaction to finally form the chiral hybrid, MGO@PNG‐CD. The chiral hybrid shows multiple advantages in that GO provides high specific surface area, Fe_3_O_4_ nanoparticles provide magneticity, PNG chains provide temperature sensitivity, and *β*‐CD units provide chiral selectivity through forming host–guest inclusion complex. Furthermore, after enantioselective adsorption of tryptophan enantiomers, easy isolation of the chiral hybrid from mixed solution can be realized simply by using external magnetic field. Owing to the pH‐sensitivity of PNG polymer chains, desorption can be implemented with much ease.^[^ ^105^ ^]^

The above preparation strategy offers a versatile platform for designing and developing chiral nanohybrids derived from graphene, cyclodextrins, and stimuli‐responsive polymer chains.^[^ ^105^ ^]^ Aside from graphene and cyclodextrin, other material also can be combined with the hybrid for developing advanced functional architectures with multicomponents, hierarchical structures, and excellent comprehensive properties, e.g., chiral hybrids constructed by carbon nanotube, RGO, and *β*‐CD toward electrochemically recognition of phenylalanine enantiomers;^[^ ^106^ ^]^ electrochemical sensors containing 3D graphene layers and hydroxypropyl‐*β*‐cyclodextrin units for recognition of tryptophan enantiomers.^[^ ^107^ ^]^ In particular noticeably, the existence of magnetic nanoparticles facilitates the chiral hybrids to be easily restored for further recycling use. Otherwise, it is an intractable task to isolate the chiral hybrids after use. Accordingly, the preparation methodology dealing with introduction magnetic nanoparticles may be applied to the preparation of other chiral materials to be hopefully used as chiral catalysts, chiral adsorbents, chiral selectors, etc. Apart from the popularly used Fe_3_O_4_ nanoparticles, other ingredients possessing magnetic property also can be taken for constructing chiral hybrids with magneticity, e.g., Co,^[^ ^112^ ^]^ CoFe,^[^ ^113^ ^]^ and CoFe_2_O_4_ nanoparticles.^[^ ^114^ ^]^ These potential candidates offer much more opportunities for developing chiral materials with magneticity, which in turn brings advantages for the chiral hybrids, including easy isolation after use and conveniently recycling use. Compared with the analogues without magneticity, the magnetic chiral materials are superior in terms of actual use operations. This allows the magnetic chiral materials to be more competent from the viewpoint of practical uses.

Regarding the multicomponent chiral materials analyzed above, the essential underlying design strategy relies in: Graphene sheets afford large surface area while cyclodextrin units offer host–guest enantioselective interactions toward chiral analytes; the other ingredients make their corresponding contribution. for instance, magnetic particles providing magneticity;^[^ ^102^, ^105^ ^]^ and rhodamine B rendering fluorescence for the hybrid chiral materials.^[^ ^106^ ^]^ Following the design and preparation strategy, more other building blocks may be utilized to provide their functions. For example, chiral pH‐responsive polymers,^[^ ^115^, ^116^ ^]^ chiral temperature‐sensitive polymers,^[^ ^117^, ^118^ ^]^ and chiral dynamic covalent structures (e.g., Schiff base^[^ ^119^ ^]^ and borate structures^[^ ^120^ ^]^) can be combined with chiral graphene hybrids to enable them to show stimuli‐responsivity. The as‐obtained smart chiral hybrids are interesting for diverse chiral applications. These stimuli‐responsive chiral graphene materials might become an attractive research direction in near future.

#### 2.1.3 Other Small Organic Molecules

Just like cyclodextrins and the derivatives, other chiral small organic molecules can be combined with graphene, GO, and RGO through covalent bonds and/or noncovalent interactions. In this section, the cases with noncovalent interactions are introduced first, followed by those involving the formation of covalent bonds between graphene or its derivatives and chiral organic molecules. The chiral small molecules used for chiral functionalization of graphene are summarized in **Table** **3**.

**Table 3:** Overview of other small molecules used for chiral functionalization of graphene and applications of the chiral hybrid materials

Chiral small molecules	Graphene (GO, RGO)	Applications^a)^	Ref.
Betamethasone	graphene	Electrochemical chiral sensing (mandelic acid)	^[^ ^121^ ^]^
Ionic liquid	GO	Electrochemical chiral recognition (tryptophan, mandelic acid, malic acid, tyrosine)	^[^ ^122^ ^]^
Maltodextrin	GO	Chiral stationary phase for separation (nefopam, amlodipine, citalopram hydrobromide, econazole, ketoconazole, cetirizine hydrochloride)	^[^ ^123^ ^]^
Naphthalenediimide	RGO	Cell bioimaging	^[^ ^124^ ^]^
Liquid crystalline surfactant	RGO	–^b)^	^[^ ^125^ ^]^
BINOL	GO	Chiral catalyst (addition of diethylzinc to aromatic aldehyde)	^[^ ^126^ ^]^
(*S*)‐(+)‐2‐Pyrrolidinemetnalol	graphene	Chiral stationary phase for separation (ibuprofen, thalidomide)	^[^ ^127^ ^]^
*R*‐mandelic acid	GO	Electrochemical chiral recognition (amino propanol)	^[^ ^55^ ^]^
2,3‐Diaminopropionic acid	GO	Hemostatic material	^[^ ^56^ ^]^
Chiral 2,2ʹdibromo‐9,9 ʹ ‐bianthracene (DBBA)	Graphene nanoribbons	–^b)^	^[^ ^128^ ^]^

^a)^ In the brackets, the corresponding chiral analytes involved in chiral applications are presented

^b)^ No application is reported.

Jabbari and co‐workers developed a chiral biosensor using betamethasone as chiral selector through multilayered electrochemical deposition of betamethasone, overoxidized polypyrrole, and graphene sheets.^[^ ^121^ ^]^ The chiral hybrid showed electrochemical enantiorecognition for mandelic acid enantiomers. Also for the purpose of electrochemical sensing, Kong et al. synthesized a chiral ionic liquid and applied it as an electrolyte to incorporate with GO sheets for preparing chiral hybrid.^[^ ^122^ ^]^ The chiral hybrid was subsequently used as chiral modifier to construct an electrochemical sensor based on GCE, which can enantioselectively distinguish several racemic enantiomers including tryptophan, mandelic acid, malic acid, and tyrosine enantiomers, according to the response of peak current resulting in different forms. Apart from being used as chiral modifiers for preparing electrochemical sensors, chirally functionalized graphene can be taken for other chiral applications. Du et al. used maltodextrin to modify GO and the chiral product was used as chiral stationary phase in open‐tubular capillary electrochromatography for chiral separation of six chiral drugs, i.e., nefopam, amlodipine, citalopram hydrobromide, econazole, ketoconazole, and cetirizine hydrochloride.^[^ ^123^ ^]^ Compared with the pristine silica capillary, the resolution outcomes using the silica capillary after chiral modification were markedly improved. In order to bioimage prostate cancer cells, Mirabello and co‐workers prepared GO/chiral naphthalenediimide chiral nanohybrids, which showed promising applications in biosensing and bioimaging fields.^[^ ^124^ ^]^ Cong and co‐workers modified RGO sheets with a chiral liquid crystalline surfactant through noncovalent interactions, more specifically, through *π*–*π* interactions occurring between RGO sheets and the surfactant molecules.^[^ ^125^ ^]^ This study significantly expands the candidates used as chiral modifiers for functionalizing graphene. More unprecedented properties and promising uses are anticipated from the unique chiral hybrids consisting of chiral liquid crystalline structures.

It has been definitely shown that covalent bonds can be employed for chiral functionalization of graphene, as demonstrated in the cases using amino acids and cyclodextrins discussed above. Yan group^[^ ^126^ ^]^ first prepared GO sheets bearing acyl chloride reactive functional groups, and then the GO derivative reacted with chiral NH_2_‐BINOLs forming amide structures (BINOL, 1‐(2‐hydroxynaphthalen‐1‐yl)naphthalen‐2‐ol). The chiral product was further treated with Ti(O*^i^*Pr)_4_ (tetraisopropyl titanate) to generate GO‐BINOL‐Ti chiral complex catalyst, which displayed good reactivity (99%) and modest enantioselectivity (45% e.e.) in the asymmetric addition reaction of diethylzinc to aromatic aldehydes.^[^ ^126^ ^]^ Serov and co‐workers functionalized mesoporous 3D graphene with tetracyanoethylene oxide and (*S*)‐(+)‐2‐pyrrolidinemethanol. The chiral product was used as chiral stationary phase for chromatographic separation, demonstrating pharmaceutical grade chiral separation of model ibuprofen and thalidomide racemic mixtures.^[^ ^127^ ^]^ Li group modified graphene using *R*‐mandelic acid‐linked calix[4]arene, which showed highly sensitive chiral recognition toward amino propanol in serum.^[^ ^55^ ^]^ Wang et al. reported a graphene sponge used for hemostasis, in which the chiral graphene material was prepared by crosslinking graphene with diaminopropionic acid.^[^ ^56^ ^]^

In a very recent publication, de Oteyza and co‐workers succeeded in transferring axial molecular chirality through a sequence of on‐surface reactions to prochiral graphene nanoribbons.^[^ ^128^ ^]^ All these studies clearly show that a great number of small organic molecules can be potentially used as chiral sources for chiral modification of graphene family materials, and the resulting chiral hybrids are worthy to be continuously exploited for diverse applications. The substantial number of studies also evidence the significant importance of chiral graphene materials. According to specific needs, suitable chiral sources in particular the natural “chiral pool”^[^ ^129^, ^130^, ^131^, ^132^ ^]^ can be utilized to develop chiral hybrids via reasonable integration with graphene and the derivatives. The “chiral pool” affords numerous possibilities for design and exploitation of novel advanced functional chiral materials.

### 2.2 Natural Biomacromolecules as Chiral Source

Natural biomacromolecules generally possess stable physicochemical properties, good biocompatibility and rich functional groups, which provide many opportunities for further modification and regulation of their properties.^[^ ^133^ ^]^ Among the commonly used biomacromolecules, polysaccharides are the most widely used category for functionalization of graphene. Amylose,^[^ ^134^ ^]^ cellulose and derivatives,^[^ ^135^, ^136^, ^137^ ^]^ chitosan,^[^ ^138^, ^139^ ^]^ and even two or more polysaccharides in combination^[^ ^140^ ^]^ are widely used for constructing graphene‐based chiral materials. The natural biomacromolecules used for chiral functionalization of graphene and the corresponding chiral applications of the resulting chiral hybrids are presented in **Table** **4**.

**Table 4:** Overview of biomacromolecules used for chiral functionalization of graphene and the applications of the chiral composite materials

Chiral biomacromolecules	Graphene (GO, RGO)	Application^a)^	Ref.
Amylose	RGO	Chiral detection (tryptophan)	^[^ ^134^ ^]^
Cellulose	RGO	Chiral stationary phase for separation (nine pairs of benzene‐containing enantiomers^b)^)	^[^ ^135^ ^]^
Carboxymethyl cellulose + Cu(II) coordinated with *β*‐CD	RGO	Electrochemical chiral detection (tryptophan)	^[^ ^136^ ^]^
Cellulose nanocrystals	GO	Self‐assembled chiral smectic structure	^[^ ^137^ ^]^
Chitosan	Graphene	Electrochemical chiral sensor (DOPA)	^[^ ^138^ ^]^
Chitosan	RGO	Electrochemical chiral detection (tryptophan)	^[^ ^139^ ^]^
Chitosan/sodium alginate	Graphene/carbon nanotube	Electrochemical chiral detection (tryptophan)	^[^ ^140^ ^]^
Bovine serum albumin (BSA)	GO	Chiral stationary phase for separation (tryptophan, threonine)	^[^ ^141^ ^]^
Human serum albumin (HSA)	GO	Chiral stationary phase for separation (nine pairs of chiral enantiomers)	^[^ ^142^ ^]^
Human serum albumin (HSA)	GO	Electrochemical chiral sensor (tryptophan)	^[^ ^143^ ^]^
*γ*‐Globulin	GO	Electrochemical chiral discrimination (mandelic acid)	^[^ ^144^ ^]^
Enzyme	Graphene	Chiral catalyst (chiral alcohols)	^[^ ^145^ ^]^
Acetylcholinesterase	RGO	Electrochemical chiral sensor (methamidophos)	^[^ ^146^ ^]^
DNA	RGO	Electrochemical chiral sensor (tryptophan)	^[^ ^47^ ^]^

^a)^ In the brackets, the corresponding chiral analytes involved in chiral applications are presented

^b)^ The nine pairs of enantiomers include ibuprofen, trans‐stilbene oxide, 2‐phenylcyclohexanone, praziquantel, propranolol, *R*,*S*‐equol, ketoconazole, benzoin, and quinidine.

#### 2.2.1 Polysaccharides

**Figure 7:**
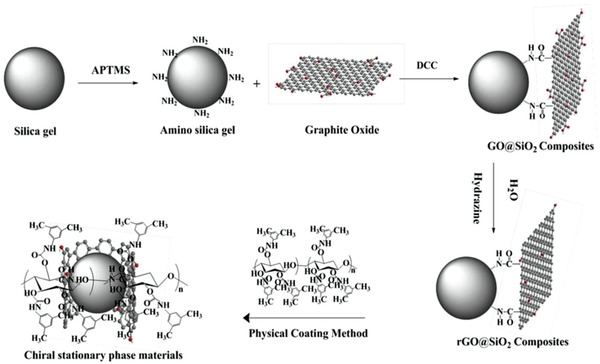
The preparation of cellulose coated rGO@silica. APTMS, (3‐aminopropyl)trimethoxysilane; DCC, *N*,*N*‐dicyclohexylcarbodiimide. Reproduced with permission.^[^ ^135^ ^]^ Copyright 2018, Wiley‐VCH.

In most cases, polysaccharides are attached onto graphene sheets by noncovalent interactions. Taking the work from Li et al. as example,^[^ ^135^ ^]^ they first modified silica core by introducing amino groups on its surface. The amino groups subsequently underwent amidation reaction with the carboxyl groups on GO, followed by the reduction of GO with hydrazine to provide RGO@silica hybrid. Finally, a cellulose derivative, i.e., cellulose tris(3,5‐dimethylphenylcarbamate) was coated on the RGO@silica hybrid. The major procedure is illustratively presented in **Figure** **7**.^[^ ^135^ ^]^ The eventually obtained chiral composite was used as stationary phase in chiral HPLC. In this work, the cellulose derivative was physically coated on reduced GO sheets, in which the phenyl groups preintroduced around cellulose backbones are considered forming hydrophobic interaction and *π*–*π* stacking with graphene skeleton. Apart from cellulose, other widely commercialized polysaccharides, e.g., starch (in particular amylose), chitosan, alginate, and hyaluronic acid, also can be taken as alternatives for combining with graphene and its derivatives, since these natural polysaccharides can be easily modified with compounds containing phenyl moieties, and especially these polysaccharides are chiral and hydrophilic.^[^ ^147^, ^148^, ^149^, ^150^ ^]^ Once integrated with graphene family materials, they not only render graphene with chirality, but also favorably assist graphene layers in keeping dispersion state. In other words, the biomacromolecules helpfully prevent graphene sheets from accumulation, due to the hydrophilicity and steric hindrance of biomacromolecules.

**Figure 8:**
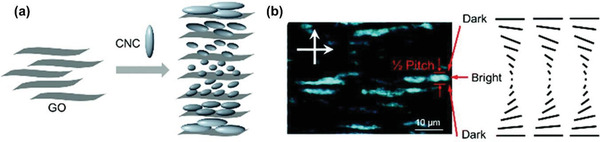
Schematic drawing of a) the formation of a chiral smectic structure in the CNC/GO composite colloid and b) the optical image of CNC/GO with schematic illustration. Reproduced with permission.^[^ ^137^ ^]^ Copyright 2017, Royal Society of Chemistry.

Cellulose nanocrystals (CNCs) have become one of the most vibrant research fields in particular in advanced functional materials. In chiral materials research area, CNCs have attracted much attention. They are predominantly used to fabricate chiral optical materials due to their ability to form liquid crystal structures.^[^ ^151^ ^]^ CNCs are also employed as chiral source for developing chiral hybrid particles^[^ ^152^ ^]^ and chiral stationary phase for chiral separation.^[^ ^153^ ^]^ Zhu et al. fabricated a chiral smectic structure assembled from graphene and CNCs.^[^ ^137^ ^]^ As illustratively shown in **Figure** **8**, the typical 2D material (GO layer) and the typical 1D biomass‐derived material (CNCs) coassembled, thereby constructing a chiral smectic structure. It was found that the formation of such a distinctive structure strictly depends on the ratio of GO/CNCs and their concentration. The study opens up a new way for developing mesoscopic materials exhibiting tunable physical properties, for instance optical metamaterials with potential applications in optical modulation and mechanochromic sensors. This work from Zhu group^[^ ^137^ ^]^ also offer insights into 1D materials like CNCs. It may also stimulate new ideas since, aside from CNCs derived from cellulose, other nano‐ and microarchitectures also can be employed in order to chirally functionalize graphene and to construct advanced functional materials. The nano‐ and microscaled architectures include starch‐based nanoparticles, chitin and chitosan‐based nanocrystals. Stimulated by the growing requirements for sustainable materials, these biomass‐based architectures provide a lot of opportunities for accomplishing the purposes.

#### 2.2.2 Proteins

**Figure 9:**
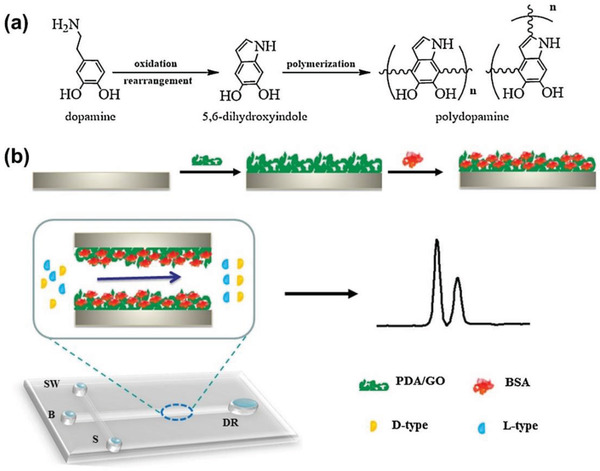
a) Schemes of simultaneous polymerization of dopamine and b) the mechanism of BSA immobilized on the PDMS microchannel through the PDA/GO layer and the chip‐based OT‐CEC system for chiral analysis using PDA/GO/BSA stationary phase integrated with in‐channel electrochemical detection. The sample reservoir (S) was filled with the mixture of enantiomers solution. Separation reservoir (B) and injection waste reservoir (SW) were filled with running buffer. Reproduced with permission.^[^ ^141^ ^]^ Copyright 2013, Elsevier.

Apart from polysaccharides, proteins constitute the second largest group of biomacromolecules that have been frequently employed for preparing chiral hybrids via integrating with graphene family materials. The integration can be realized through noncovalent interactions and/or covalent bonds. Qiu lab first deposited polydopamine–graphene oxide (PDA/GO) on a poly(dimethylsiloxane) (PDMS) microfluidic chip, and then conjugated bovine serum albumin (BSA) on it.^[^ ^141^ ^]^ The resulting chip‐based chiral hybrid was used as enantioselective open‐tubular capillary electrochromatography (OT‐CEC). As schematically shown in **Figure** **9**, in the first coating layer (PDA/GO), PDA makes it easy to immobilize the coating in the microchannel, while GO offers large surface and excellent biocompatibility that can incorporate much more biomolecules and well maintain their biological activity. In addition, GO makes the surface morphology much rougher, which is favorable for enhancing the subsequent loading capacity of proteins in the microchannels and increasing sample capacity of OT‐CEC columns. BSA was taken as a model and immobilized in the microchannel to fabricate a protein‐stationary phase. Compared with the pristine PDMS microchannels, the as‐modified surface exhibited much better wettability, more stable electroosmotic mobility, and less nonspecific adsorption. Separating effects toward amino acids and chiral dipeptide demonstrate that the chirally modified OT‐CEC columns own good enantioselectivity. The study using PDA/GO coating as a versatile platform for facile conjugation with proteins establishes a new processing technique to manufacture functional surfaces designed on microfluidic chips.

**Figure 10:**
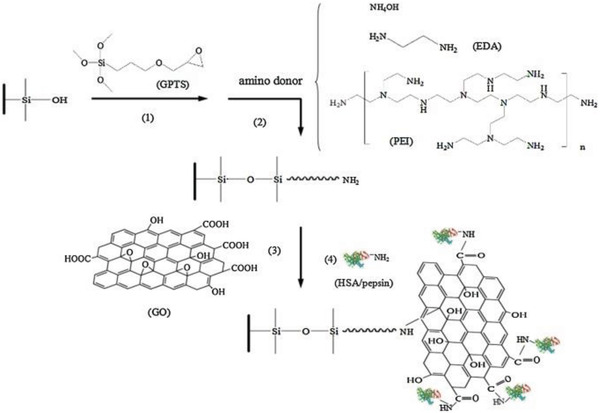
Schematic presentation of the procedure for preparing GO‐modified affinity capillary silica monoliths. Three types of amino donors were used to investigate the effects of spacer arm (connecting silica substrate and GO sheets) on chiral differentiation ability of the obtained chiral hybrid materials. Reproduced with permission.^[^ ^142^ ^]^ Copyright 2016, Elsevier.

In the above study, BSA was noncovalently immobilized on GO, taking advantage of the large surface area of GO sheets. Proteins can be also efficiently attached on substrates alternatively through chemical reactions. In the study from Li and co‐workers,^[^ ^142^ ^]^ affinity capillary monoliths were first modified with GO and then used HAS or pepsin as chiral selectors for the purpose of chiral separation (**Figure** **10**). In the process of coating GO layer, three types of amino donors were examined (ammonium hydroxide, NH_4_OH; ethanediamine, EDA; and polyethyleneimine, PEI) to explore the influence of spacer arm on enantio‐separation effect.^[^ ^142^ ^]^ Amongst the resulting chiral material systems, HAS‐GO‐EDA system exhibited the best chiral recognition ability; the as‐prepared columns demonstrated satisfactory column‐to‐column, run‐to‐run, and interday repeatability. HAS‐GO‐EDA based column also showed higher chiral selectivity toward nine pairs of enantiomers (phenylalanine, azelastine, warfarin, ibuprofen, salbutamol, chlortrimeton, propranolol, nefopam, and traptophan), when compared with the column without modification by GO. The results indicate that the stacking between biomacromolecules, GO sheets, and the silica substrate exert remarkable influence on the enantio‐discernibility of the chiral product. The stacked architecture directly affects the interactions between the chiral selecting sites and the chiral analytes to be differentiated.

GO‐functionalized affinity capillary monoliths have promising potential for chiral applications. Considering that proteins and chitosan have abundant amino groups, the route developed by Li and co‐workers may be applicable to these types of biomacromolecules and even chiral small molecules containing amino groups. Also notably, monolithic materials show advantages of easy preparation and high porosity.^[^ ^154^, ^155^ ^]^ Such chiral materials may be exploited as chiral sponges used in some specific situations, such as capturing chiral contaminants from wastewater for restoring chiral compounds and simultaneously solving chiral pollution in environment.^[^ ^156^, ^157^ ^]^ The chiral monolith also may be developed as supports for chiral catalysts, due to their high porosity which is favorable to solve the problem of mass transfer in routine catalytic reaction systems.

In other studies, HAS, GO and indium tin oxide (after modification with 3‐aminoproyltriethoxysilane) based electrochemical biosensor was developed for chiral discrimination of tryptophan;^[^ ^143^ ^]^ glassy carbon electrode modified with RGO and *γ*‐globulin was fabricated for enantioselective differentiation of mandelic acids;^[^ ^144^ ^]^ and acetylcholinesterase/GO based sensors were constructed for electrochemical chiral detection of chiral molecules (methamidophos).^[^ ^146^ ^]^ The studies using various proteins to combine with graphene for diverse chiral applications have paved a solid foundation for further exploiting chiral materials of the kind. These novel chiral hybrids show a wide range of actual application potentials toward multiple research areas and industries, like chiral separation, asymmetric catalysis, chiral sensing and recognition, chiral adsorption, etc.

#### 2.2.3 Other Biomacromolecules

Besides the widely explored polysaccharides and proteins as summarized above, some other biomacromolecules also have potentials to be exploited for developing chiral materials. For instance, DNA was adopted as a chiral selector and immobilized in graphene‐based substrate, for the purpose of enantioselectively electrochemical recognition of tryptophan.^[^ ^47^ ^]^ Nonetheless, the studies using DNA and other types of biomacromolecules, with the exception of polysaccharides and proteins, are still quite limited in both variety and number. Owing to their unique structures and properties, the kind of biomacromolecules like DNA may perform distinctive functions when combined with graphene, which cannot be achieved in polysaccharides and proteins, much less chiral small molecules. With DNA as representative, the double helix structure may allow DNA‐based chiral hybrids to exhibit some fascinating properties. Accordingly, using DNA and other biomacromolecules that have not been explored extensively so far to develop novel chiral hybrid materials is expected to bring unique opportunities for chirality associated research areas. In brief, natural biomacromolecules can make much more contribution than just serving as chiral source. Their rational conjugation with graphene is a research subject deserving much more attention. The studies might not only lead to series of novel chiral hybrid materials, but also possibly provide some new insights into the intriguing chiral stereo‐architectures formed in biomacromolecules, for instance, double helices of DNA and *α*‐helices and *β*‐sheets of proteins. The endeavors may also stimulate rapid advancements of artificial helical polymers,^[^ ^158^, ^159^ ^]^ which are designed and developed by imitating the natural analogues, as to be investigated in the next section.

### 2.3 Synthetic Polymers as Chiral Source

Up to date, a myriad of composite materials has been explored based on graphene and polymers. The composites have been investigated in a wide range of research areas and industries.^[^ ^160^, ^161^, ^162^ ^]^ In spite of the numerous materials consisting of graphene and polymer already created so far, the chiral analogues are quite limited in both type and number. Muthusamy group^[^ ^163^ ^]^ first prepared a chiral diamine starting from l‐phenylalanine; the diamine underwent polymerization with 3,3’,4,4’‐benzophenone tetracarboxylic dianhydride by thermal imidization method, thereby forming a chiral polyimide. The polyimide was then blended with GO to form a chiral GO/polyimide nanocomposite, aiming at investigating the effects of GO on the thermal, electrical, and morphological properties of the polymer. GO was found to uniformly dispersed in the chiral polyimide matrix; the dispersed phase formed strong bonds with the polymer matrix due to GO grafted with polyimide. The existence of GO improved the glass transition temperature of the polyimide from 225 °C (pristine polyimide) up to 251 °C (polyimide in the nanocomposite). The as‐obtained nanocomposite showed remarkable improvement in thermal, electrical, and mechanical performance, which is considered being derived from the good interfacial bonding between the two phases and the excellent dispersion of GO in the polymer matrix. Especially noticeably, biological studies show that the chiral nanocomposite exhibited antimicrobial effects against Gram‐positive and Gram‐negative bacteria. The chiral nanocomposite deserves more exploration. The antimicrobial property, together with the intrinsic easy processing ability of polymers, renders the chiral nanocomposite with promising applications as biological materials. Generally speaking, polyimides have better thermal and mechanical properties than common polymers and so have found practical applications in aerospace, electrical industries, automobile industries, fuel cells, etc.^[^ ^164^, ^165^ ^]^ Affording polyimides with chirality makes them more interesting and expands their applications to chiral areas. Combination with graphene further favorably improves their integral properties and considerably extends their potential applications.

Natural biomacromolecules form uniform chain conformations, like double helix in DNA, *α*‐helical and *β*‐sheet structures in proteins, helical structures in polysaccharides, which are closely correlated with noncovalent interactions.^[^ ^147^, ^148^, ^150^ ^]^ The regular stereo‐architectures and conformational chirality of biomacromolecules play vital roles for organisms to keep normal functions and biological processes. To acquire deep understanding of the chiral biomacromolecules, scientists have synthesized a series of chiral helical polymers and helical architectures imitating the natural counterparts.^[^ ^158^, ^159^ ^]^ The typical artificial chiral helical polymers include polyacetylenes,^[^ ^166^ ^]^ polyisocyanides,^[^ ^167^ ^]^ polycarbodiimides,^[^ ^168^ ^]^ poly(quinoxaline‐2,3‐diyl)s,^[^ ^169^ ^]^ and others.^[^ ^158^, ^159^ ^]^ If combing synthetic helical polymers with graphene, the resulting chiral hybrids may incorporate the respective advantages of chiral polymers and graphene materials. Chen and co‐workers^[^ ^46^ ^]^ integrated poly(l‐glutamic acid sodium) (PLGA) and GO to fabricate chiral membrane. The synthetic chiral polymers used for chiral functionalization of graphene are summarized in **Table** **5**. Among the synthetic chiral polymers, those being able to adopt helical conformations generally exhibit unique superiority compared with the ones without helical conformations, because of the chiral amplification effect frequently observed in chiral helical polymers.^[^ ^170^ ^]^

**Table 5:** Overview of synthetic polymers used for combining with graphene and application of the chiral composite materials

Chiral polymers	Graphene (GO, RGO)	Properties/Application^a)^	Ref.
Polyimide	GO	Antimicrobial	^[^ ^163^ ^]^
Poly(l‐glutamic acid sodium) (PLGA)	GO	Chiral membrane for separation (DOPA)	^[^ ^46^ ^]^
Poly(phenyl isocyanide)s	GO	Inducing enantioselective crystallization (alanine)	^[^ ^171^ ^]^
Acryloyl *N*‐*β*‐l‐aspartyl‐l‐phenylalanine dimethyl ester	GO	Electrochemical chiral sensing (monosaccharide)	^[^ ^172^ ^]^
Liquid crystal oligomer	RGO	Enhancing photocatalytic degradation of TiO_2_	^[^ ^173^ ^]^
Substituted polyacetylene	Graphene	Optically active hybrid	^[^ ^174^ ^]^
Substituted polyacetylene	RGO	Chiral monolith	^[^ ^175^ ^]^
Substituted polyacetylene	GO	Chiral monolith for adsorption (alanine, leucine, phenylalanine, phenethylamine, Boc‐alanine)	^[^ ^176^ ^]^
Substituted polyacetylene	GO	Chiral nanocomposite	^[^ ^177^ ^]^
Substituted polyacetylene	GO	Chiral hybrid microspheres	^[^ ^178^ ^]^
Substituted polyacetylene	GO	Chiral adsorbent (phenylalanine, DOPA)	^[^ ^179^ ^]^
Substituted polyacetylene	GO	Chiral hybrid nanoparticles, inducing enantioselective crystallization (alanine)	^[^ ^180^ ^]^
Substituted polyacetylene	GO	Chiral hybrids, inducing enantioselective crystallization (alanine)	^[^ ^181^ ^]^
Substituted polyacetylene	GO	Chiral nonspherical particles, inducing enantioselective crystallization (phenylalanine)	^[^ ^182^ ^]^
Substituted polyacetylene	RGO	Chiral monolith, inducing enantioselective crystallization (alanine)	^[^ ^183^ ^]^

^a)^ In the brackets, the corresponding chiral analytes for chiral applications are presented.

**Figure 11:**
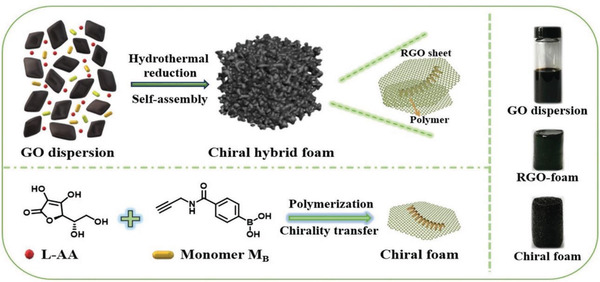
One‐step method to combine chiral helical polyacetylene and graphene. GO was reduced with l‐AA, which simultaneously acted as chiral source for forming chiral helical polymer. Reduced GO and the chiral polymer coassembly to form the chiral foam. Reproduced with permission.^[^ ^175^ ^]^ Copyright 2019, Wiley‐VCH.

Among the artificial chiral helical polymers, substituted polyacetylenes have been substantially investigated and may serve as representative. Deng group introduced aromatic pyrene moieties in the pendant chains around polyacetylene backbones and integrated the polymer with graphene.^[^ ^174^ ^]^ Owing to the *π*–*π* interactions between pyrene groups and graphene skeleton, the polyacetylene chains can be immobilized on graphene layers. For the copolymers derived from acetylenic monomer bearing pyrene substituents and chiral acetylenic monomers, they formed helical structures with predominant one‐handed helicity and thus possessed optical activity. After combined with graphene, the chiral helical copolymers retained their predominant helicity and thereby afforded the graphene/polymer hybrid with optical activity. This study opens up a route for chiral functionalization of graphene with chiral helical substituted polyacetylenes. On the basis of the study,^[^ ^174^ ^]^ Deng group further established a one‐step process for preparing chiral hybrid composed of graphene and helical polyacetylene.^[^ ^175^ ^]^ The preparation strategy is illustratively presented in **Figure** **11**.

In Figure 11, three originally independent processes were combined in one single process. The three processes included 1) reduction of GO for forming RGO; 2) self‐assembly of RGO layers to construct graphene foam; and 3) helix‐sense‐selective polymerization (HSSP) of achiral monomer to generate chiral helical polymer. As far as HSSP is concerned, it is widely explored in asymmetric polymerization for preparing chiral helical polymers starting from achiral monomers.^[^ ^184^, ^185^ ^]^ Diverse strategies can be taken to induce the otherwise racemic helical polymer chains to form one homohelicity or one predominant helicity. These strategies include using chiral catalyst, chiral monomer‐achiral monomer copolymerization, chiral field, and chiral postinduction.^[^ ^186^, ^187^ ^]^ In the study (Figure 11),^[^ ^175^ ^]^ achiral acetylenic monomer (M_B_) underwent HSSP with l‐ascorbic acid (l‐AA) as chiral source. Indeed, l‐AA is widely used as reducing agent for the reduction of GO. In this work, l‐AA played double roles simultaneously, i.e., as reducing agent for the reduction of GO and as chiral source for performing HSSP of the achiral monomer. Chirality transfer from l‐AA to the polymer chains was realized via the boric acid group in the monomer and polymer side chains, which can form borate with l‐AA. This study is interesting since the preparation methodology may be applicable to other achiral monomers, from which chiral graphene hybrids are also anticipated. From the viewpoint of the chiral product, it is also attractive because of the advantages of optical activity and high porosity, respectively, derived from chiral helical polyacetylene and graphene.

Using achiral helical polymers to incorporate with graphene also can lead to chiral hybrids. The preparation process involves the strategy of chirality transfer. Deng group first carried out chiral functionalization of GO through covalently grafting chiral 1‐phenylethylamine (*R*‐ and *S*‐PEA) on GO layers.^[^ ^177^ ^]^ Then the achiral polymer and chiral GO were co‐precipitated from a same dispersion mixture, during which helix‐sense‐selective precipitation occurred to the initially achiral helical polymer chains. Moreover, the polymer chains aggregated into particles physically attached on the GO layers. The chiral helical polymer chains induced by chiral PEA afforded the hybrid with optical activity. The polymer particles were attached onto GO layers in virtue of noncovalent interactions. The study shows advantages in two aspects. The first one is the chirality transfer from the chiralized GO to the initially achiral helical polymer chains, which induced the racemic helical polymer to adopt one predominant helicity. The second one is the formation of chiral polymer particles by precipitation process. The methodology of helix‐sense‐selective precipitation is not only a conceptually new strategy, but also opens an alternative route for fabricating chiral polymer particles.

As mentioned above, chiral helical polyacetylene chains and the particles thereof were adhered onto GO layers by noncovalent effects.^[^ ^177^ ^]^ From the viewpoint of practical uses, the adhesion bond between the two components, i.e., polymeric material and GO, may be not strong enough for the hybrid to keep intact. To solve the limitation, the same group prepared a series of chiral hybrids starting from helical polyacetylenes and graphene, in which the ingredients were connected via covalent bonding. The prepared chiral materials include 1) chiral hybrid particles prepared by suspension polymerization using GO as stabilizer;^[^ ^178^ ^]^ 2) chiral monolithic adsorbent prepared by solution polymerization;^[^ ^176^ ^]^ 3) chiral helical polymer chains grafted on GO in which the polymer can reversibly undergo conformational transformation between extended free chains and particles, bio‐imitating jellyfish.^[^ ^179^ ^]^ These investigations remarkably expand the types of the chiral materials from irregular, bulky morphology formed via solution polymerization to the ones possessing regular and hierarchical structures formed from suspension polymerization and co‐precipitation processes.

**Figure 12:**
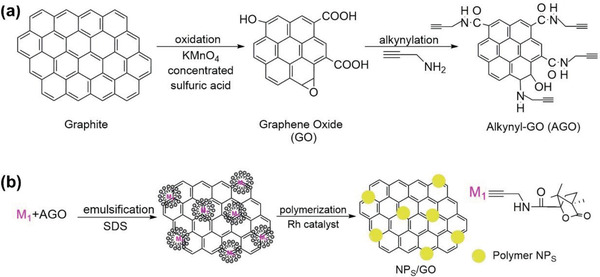
Chiral helical polymer chains grafted on GO forming nanoparticles covalently anchored on GO sheets. Reproduced with permission.^[^ ^180^ ^]^ Copyright 2014, American Chemical Society.

Up to now, Deng group has prepared a series of optically active particles derived from chiral helical polymers.^[^ ^188^, ^189^, ^190^, ^191^ ^]^ Polymer particles, in particular nanoscaled particles, tend to accumulate due to their small dimensions. When homogeneously attached on a substrate, the nanoparticles may keep a good dispersion state. To justify this assumption, Deng group built diverse approaches for preparing chiral hybrids constructed by GO and nanoparticles derived from chiral helical polymer chains. As illustrated in **Figure** **12**,^[^ ^180^ ^]^ GO was first prepared and then polymerizable acetylenic moieties were introduced on GO sheets via amidation reaction between the carboxyl groups on GO and propargylamine, resulting in alkynyl‐GO (AGO). In the next step, the chiral monomer (M_1_) underwent emulsion polymerization with the assistance of emulsifier; the preintroduced acetylenic groups on GO took part in copolymerization, in situ forming nanoparticles that were linked with GO by covalent bonding.

**Figure 13:**
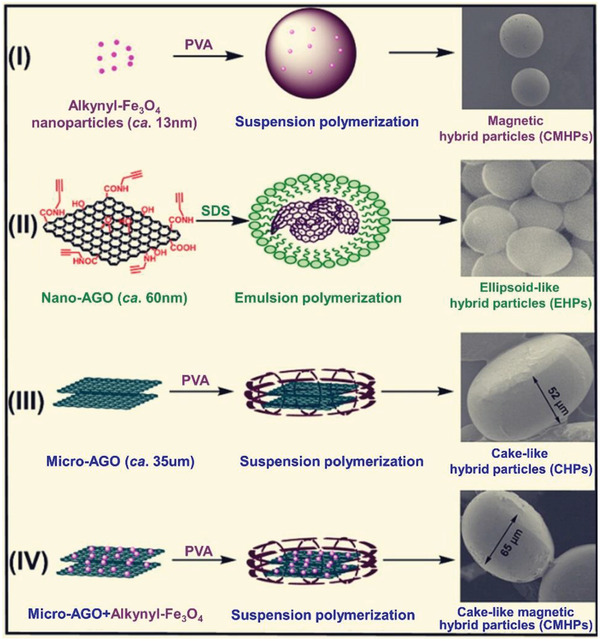
Construction of spherical and nonspherical chiral hybrid particles based on chiral helical polymer, magnetic nanoparticles and GO. AGO, alkynyl‐graphene oxide. Reproduced with permission.^[^ ^182^ ^]^ Copyright 2016, American Chemical Society.

Deng et al. found that if acetylenic monomer underwent polymerization in the presence of AGO, chiral helical polymer chains were generated and covalently connected to GO, thanks to the preintroduced polymerizable propargyl moieties which participated in the polymerization. Moreover, the chiral polymer chains retained freely extending state in the polymer's good solvent.^[^ ^181^ ^]^ In another work, the same group prepared spherical and nonspherical chiral hybrid particles using chiral helical polyacetylene and GO. If Fe_3_O_4_ nanoparticles are present, chiral hybrid particles with magneticity and nonspherical morphology can be obtained, as illustratively presented in **Figure** **13**.^[^ ^182^ ^]^ The shape of the resulting chiral hybrid particles is dependent on the size of the “hard” additives, i.e., GO sheets. With smaller GO layers (≈60 nm, nano‐AGO), ellipsoid‐like particles were produced; larger GO layers (≈35 µm, micro‐AGO) led to cake‐like particles. If Fe_3_O_4_ nanoparticles were present together with micro‐MGO, cake‐like magnetic chiral hybrid particles were formed.

The studies^[^ ^180^, ^181^, ^182^, ^183^ ^]^ established approaches for constructing chiral hybrid materials making use of graphene and chiral polymers. The resulting chiral hybrids rationally combined the advantages of chiral polymers (optical activity and stimuli‐responsivity) and the advantages of graphene (nano‐ and microscale, high specific surface area, etc.) in a single material. If further combined with other matters such as Fe_3_O_4_ particles, chiral hybrids possessing more appealing properties can be fabricated thereof. The related preparation methodologies can be taken as versatile and robust platforms, potentially leading to a great number of unprecedented chiral hybrids and even hybrid materials without chirality. At this point, it is worthy to mention that Dong and co‐workers reported another process to attach substituted polyacetylene to graphene through nitrene chemistry reaction.^[^ ^192^ ^]^ The organic–inorganic hybrids are anticipated to find uses that cannot be realized using the routine materials. Smart materials, architectures, and devices also are expected from future efforts along the research direction.

Apart from chiral helical polyacetylenes, Zhu and co‐workers prepared chiral hybrid materials using GO and chiral helical polyisocyanides.^[^ ^171^ ^]^ Just like chiral helical polyacetylenes discussed above, the formation of hybrids with GO layers cannot prevent the polyisocyanide chains from forming chiral helical structures. The helical polymer endowed the hybrid materials with intriguing optical activity. Accordingly, the chiral hybrids were further exploited as chiral selectors in chiral separation. The studies above evidently demonstrate that other chiral polymers in principle can be used to construct chiral hybrids with graphene. Considering the large number of chiral polymers, the types and number of potential chiral hybrids prepared following similar strategies will be numerous. Such studies may provide new findings, properties, and applications that have never been disclosed in routine materials. Also notably, conjugated polymers like substituted polyacetylenes and polyisocyanides mentioned above usually show unsatisfying processing capability. Combining them with graphene may help circumvent this trouble to some degree, since the chiral hybrids (e.g., particles and monoliths) can be used directly. In addition, graphene family materials generally have relatively higher thermal stability. This merit is also desirable for improving thermo‐properties of synthetic polymers.

Other polymers and oligomers have also been taken to combine with graphene for preparing chiral materials. For instance, Zhu and co‐workers established an electrochemical chiral sensing method based on stimuli‐responsive copolymer and graphene, which was used to modify screen‐printed carbon electrode.^[^ ^172^ ^]^ The chiral hybrid was realized using ATRP process and then acted as a chiral recognition selector. Meanwhile, it provided a chiral signal amplification effect. The chirally modified electrode was successfully applied for discriminating glucose enantiomers in live cells. The study provides a strategy to develop sensitive, facile, and cost‐effective techniques for chiral recognition of molecules in biological processes. Zhang and coworkers realized covalent modification of RGO using chiral liquid crystalline oligomer.^[^ ^173^ ^]^ The as‐prepared chiral hybrid enhanced the photocatalytic degradation efficiency of TiO_2_. Also worthy to be mentioned is that in this study, Diels–Alder (DA) reaction was taken to modify graphene. The approach can be applicable to a series of molecules for modification of graphene. Graphene combined with chiral liquid crystals is anticipated to lead to advanced functional materials for further developing reflective displays, optical filters, and electronic and optical devices for chiral detection.

### 2.4 Metal Complexes as Chiral Components

Metal and ligands together can be taken as a unit to be anchored on graphene. Chiral metal complex systems have been of much interest especially in the field of catalysis.^[^ ^193^, ^194^ ^]^ By carefully searching literature, chiral Mn complexes are found to be the most widely used to combine with graphene. As far as the chiral metal complexes are concerned, they have been substantially utilized as catalysts. After combined with chiral ligands, such metal complexes can efficiently catalyze asymmetric reactions, one of the most crucial approaches toward the generation of chiral compounds. Unfortunately, routine metal complexes have some intractable weakness, especially their recovery from reaction solution system and the subsequent recycling uses. Studies show that immobilization of metal complex on supports can helpfully solve the problem. Graphene offers such a robust support for carrying metal complexes. So far there have been a variety of processes established for attaching metal complexes on graphene layers. The chiral metal complexes immobilized on graphene family materials are summarized in **Table** **6**.

**Table 6:** Overview of chiral metal complexes used to combine with graphene and application of the chiral composite materials

Metal complex	Graphene (GO, RGO)	Application^a)^	Ref.
[Mn(TPyP)tart]^b)^	GO	Chiral catalyst (olefin oxidation)	^[^ ^195^ ^]^
[Mn(TPyP)OAc]/l‐tartrate]^c)^	GO	Chiral catalyst (olefin epoxidation)	^[^ ^196^ ^]^
Mn‐salen complex	GO	Chiral catalyst (olefin epoxidation)	^[^ ^197^ ^]^
[Mn(L)(OH)], L = (1*R*,2*S*)‐1‐(*N*‐salicylideneamino)‐2‐indanol	RGO	Chiral catalyst (olefin epoxidation)	^[^ ^198^ ^]^
Mn‐salen complex	GO	Chiral catalyst (alkene epoxidation)	^[^ ^199^ ^]^
Mn‐salen complex	GO	Chiral catalyst (olefin epoxidation)	^[^ ^200^ ^]^
Cu‐1,10‐phenanthroline	RGO	Electrochemical chiral discrimination (tryptophan)	^[^ ^201^ ^]^
Ti‐salen complex	GO	Chiral catalyst (sulfoxidation)	^[^ ^202^ ^]^
Λ‐[Os(phen)_3_(ClO_4_)_2_]^d)^	RGO	Electrochemical sensing (binaphthol)	^[^ ^203^ ^]^
Fe‐(ferrocenyl‐schiff base)	RGO	Microwave absorption	^[^ ^204^ ^]^
Rh multicomponent catalyst	Graphene	Chiral catalyst (hydrogenation)	^[^ ^205^ ^]^
Ru‐thiourea ligand	GO	Chiral catalyst (ketone transfer hydrogenation)	^[^ ^206^ ^]^
Ni nanoparticles/chiral tartaric acid	RGO	Chiral catalyst (hydrogenation)	^[^ ^207^ ^]^
Pd nanoparticles/ cinchonidine	GO	Chiral catalyst (hydrogenation)	^[^ ^208^ ^]^
Pt nanoparticles/l‐tryptophan	RGO	Electrochemical chiral recognition (DOPA)	^[^ ^209^ ^]^
Ru–Au nanoparticles/*β*‐CD	RGO	Electrochemiluminescence chiral discrimination (proline)	^[^ ^210^ ^]^
MOF	GO	Chiral capture (2,2ʹ‐furoin, benzoin)	^[^ ^211^ ^]^
MOF	GO	Chiral capture (2,2ʹ‐furoin)	^[^ ^212^ ^]^
MOF	RGO	Electrochemical chiral discrimination (tryptophan)	^[^ ^213^ ^]^

^a)^ The corresponding asymmetric reaction and the analytes for chiral detection are presented in the brackets

^b)^ TPyP, tetra(pyridylporphyrine)

^c)^ OAc, acetate

^d)^ phen, phenanthroline.

Taking Mn complexes as representative, the preparation processes for chiral graphene hybrids can be generally classified into three major categories. 1) Chiral Mn complex is first prepared and then immobilized onto modified graphene.^[^ ^197^, ^198^, ^199^, ^200^ ^]^ 2) Mn complex is first prepared and then immobilized on premodified graphene, followed by introducing chiral components directly combined with the metal complex.^[^ ^195^ ^]^ 3) Mn complex is first prepared and immobilized on premodified graphene; then chiral components are introduced onto graphene. In the last type, the metal complex and chiral component are both directly connected with graphene, likewise resulting in heterogeneous chiral catalysts.^[^ ^196^ ^]^ Based on the three primary preparation processes stated above, other metal complexes can be alike immobilized on graphene layers, for example, Cu complex^[^ ^62^, ^201^ ^]^ and Ti complex.^[^ ^202^ ^]^ The resulting chiral hybrids were employed as chiral catalysts for triggering asymmetric reactions. In spite of the various methods for immobilizing metal complex onto graphene, new techniques are still under continuously exploitation. Min et al. first accomplished chiral functionalization of graphene, and then Ru complex was attached on to the modified graphene already containing chiral moieties.^[^ ^206^ ^]^

**Figure 14:**
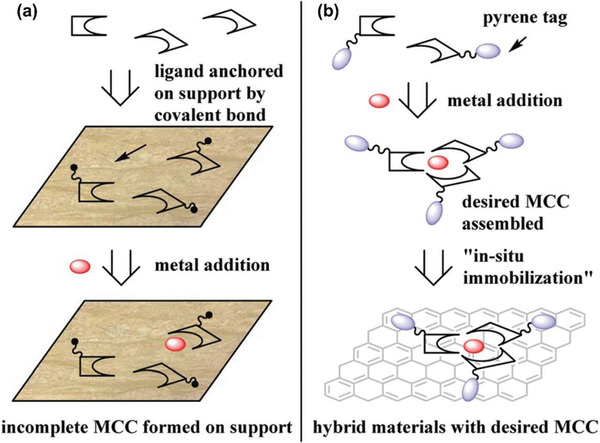
a) The traditional polymer‐supported catalyst with an incomplete MCC and b) preparation of hybrid graphene catalysts via the “in situ immobilization” strategy. MCC, multicomponent chiral catalyst. Reproduced with permission.^[^ ^205^ ^]^ Copyright 2019, Royal Society of Chemistry.

Shi et al. developed a “in situ immobilization” strategy for immobilizing Rh complex on graphene.^[^ ^205^ ^]^ Pyrene‐tagged, multicomponent chiral catalysts were anchored on graphene via *π*–*π* stacking interaction. As illustrated in **Figure** **14**, the chiral catalyst containing pyrene units was adsorbed onto graphene through *π*–*π* interaction between the pyrene units and graphene layers. The strategy shows some advantages when compared with the counterparts obtained through traditional process. The well‐defined structural features of the multicomponent chiral catalysts can be maintained entirely, concurrently avoiding the need for extra chemical modification of the moisture‐sensitive metal complex catalysts. The as‐attained hybrid chiral catalyst demonstrates excellent asymmetric induction in catalytic hydrogenation of dehydroamino acid derivatives. Moreover, the catalyst can be easily recycled without leaching toxic metal ions into the products.^[^ ^205^ ^]^

Some other metal complexes were anchored on graphene via diverse linking spacers, including Os complex^[^ ^203^ ^]^ and Fe complex.^[^ ^204^ ^]^ Different from most of the chiral metal complex catalyst hybrids developed for asymmetric catalysis, Os‐based chiral hybrid was intended for electrochemical sensing,^[^ ^203^ ^]^ while the Fe‐based chiral hybrid was intended for microwave absorption.^[^ ^204^ ^]^ Some metal complexes were immobilized on graphene in the form of metallic nanoparticles, including Ni nanoparticles,^[^ ^207^ ^]^ Pd nanoparticles,^[^ ^208^ ^]^ Pt nanoparticles,^[^ ^209^ ^]^ and Ru nanoparticles.^[^ ^210^ ^]^ Most of the chiral hybrid catalysts were developed for accomplishing asymmetric catalysis. Besides these investigations, some efforts were devoted to immobilizing metal organic frameworks (MOFs) onto graphene.^[^ ^211^, ^212^ ^]^ These chiral hybrids show advantages in high porosity and high specific surface area, so they were used for enantioselective capture purpose. The special structure of MOFs enables such chiral hybrids to be competent for chiral applications.

### 2.5 Multiple Component‐Constructed Chiral Graphene Hybrid Materials

**Figure 15:**
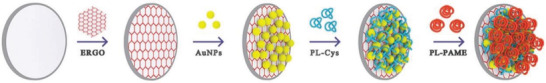
Step‐wise preparation procedure of GCE/AuNPs/PL‐Cys/PL‐PAME modified electrode (GCE, glassy carbon electrode; ERGO, electrochemically reduced graphene oxide; AuNPs, Au nanoparticles; PL‐Cys, polymer of l‐cysteine; PL‐PAME, polymer of l‐phenylalaninemethylester). The four steps were implemented by electrochemical techniques (electrochemical deposition, reduction and polymerization). Reproduced with permission.^[^ ^52^ ^]^ Copyright 2019, Springer Nature.

For constructing chiral functional materials, graphene and GO/RGO are frequently incorporated with more other building blocks, together with one or more chiral components. The resulting multicomponent chiral composites usually have complicated architectures which correspondingly endow the materials with multitude functions. Apart from 2D graphene sheets, 0D nanoparticles and 1D rod‐like building blocks are also widely adopted for fabricating chiral composites with tailored properties and functions. Akgemci et al. developed an electrochemical chiral sensor for discrimination of Trp enantiomers in aqueous medium.^[^ ^52^ ^]^ The chiral sensor was fabricated by a step‐wise self‐assembled process, as shown in **Figure** **15**. RGO, Au nanoparticles, poly(l‐cysteine), and poly(l‐phenylalanine methyl ester) were successively deposited on glassy carbon electrode. Each of the layers was deposited by electrochemical techniques including electrochemical reduction and polymerization. The as‐modified electrode showed enantiomeric differentiation ability toward Trp isomers.

In the report from Kuang et al.,^[^ ^50^ ^]^ GO and gold nanoparticles (Au NPs) underwent assembly, driven by a splint DNA strand. The DNA strand was designed with two regions at both ends, which were complementary with the DNA sequence anchored on GO and Au NPs. The prepared multicomponent hybrid successfully detected dual biomarkers in living cells. The chiral hybrid also showed good selectivity and stability. The detection strategy not only has potential utility in designing and constructing biomarkers, but also provides an avenue to allow highly accurate and reliable diagnosis of clinic diseases. Tao and co‐workers reported a simple and straightforward route established for the fabrication of hierarchical chiral materials through the assembly of GO sheets and cellulose nanocrystals (CNCs).^[^ ^51^ ^]^ The hierarchical structure of the assembled CNC/GO hybrid was preserved in solid state, facilitating electrode active SnO_2_ to be further loaded for uses in conversing and storing energy. Moreover, the assembled multicomponent structure can be further processed into film, fiber, and textile. The free‐standing SnO_2_/CNC/RGO electrodes exhibited high energy storage performance. Accordingly, the work establishes an alternative route for manufacturing new flexible energy storage devices. Controlled covalent functionalization of reduced graphene oxide can generate well‐defined bifunctional 2D nanomaterials, as reported by Haag and co‐workers^[^ ^214^ ^]^ The preparation methodology provides an alternative access to chiral surfaces and multifunctional hybrid architectures with potential uses in various areas.

## 3 Chiral Applications of Chiral Graphene Hybrid Materials

Chirality exists ubiquitous in nature. Nonetheless, the importance of chiral compounds was extensively recognized only in the second half of the 20th century.^[^ ^1^, ^215^, ^216^, ^217^ ^]^ In this section, chiral applications of the graphene‐based chiral hybrids discussed above are summarized. The chiral applications will be sub‐grouped into asymmetric catalysis, chiral detection (including chiral recognition and chiral sensing), chiral separation (also called chiral resolution in some literatures) with chromatography, membrane, enantioselective crystallization and adsorption techniques, biomedical applications (bioimaging, chiral carrier for drugs, hemostatic materials, etc.), and other chiral uses.

### 3.1 Asymmetric Catalysis

In most cases, chiral compounds can be acquired via three ways: 1) natural “chiral pool” (using naturally existing chiral substances to produce new chiral compounds);^[^ ^129^, ^130^, ^131^, ^132^ ^]^ 2) asymmetric synthesis (as to be addressed below. Ideally, only one enantiomer is generated exclusively; however, this is not true in a major part of the reactions); and 3) chiral switch (switch from the undesired enantiomer to the required one).^[^ ^2^ ^]^ At present, the primary approach toward enantiopure compounds is asymmetric reactions. Accordingly, a large number of chiral catalysts have been developed for catalyzing asymmetric reactions. Furthermore, the 2001's Nobel Prize in chemistry was awarded to the three Professors for their contribution to asymmetric catalysis.^[^ ^218^ ^]^ This event also evidences the importance of asymmetric catalysis and chiral compounds.

Graphene provides an excellent platform for creating new catalysts due to its 2D architecture and unique electrochemical properties. Among the chiral building blocks for constructing graphene based chiral material systems summarized in Section 2, proline,^[^ ^41^, ^43^, ^53^, ^58^ ^]^ enzymes,^[^ ^145^ ^]^ and metal complexes^[^ ^195^, ^196^, ^197^, ^198^, ^199^, ^200^, ^202^, ^205^, ^206^, ^207^, ^208^ ^]^ have become the most extensively used chiral species for developing chiral hybrid catalysts. To better address the issues, at this point, organocatalysis should be remarked first. The concept of organocatalysis has been recognized since the 1970s. Nonetheless, it began to flourish after the List and colleagues’ publication ≈ 30 years later.^[^ ^219^ ^]^ Direct asymmetric aldol reactions constitute one of the most substantially explored reactions with much importance in organic synthesis associated with pharmaceutical, agrochemical, and fine chemical industries.^[^ ^220^, ^221^ ^]^ Aldol reactions giving rise to C−C bond formation has played a leading role in asymmetric catalysis field. In this type of reactions, using simple building blocks can synthesize more complicated, biologically active species. Since the pioneering work of List et al. in 2000,^[^ ^219^ ^]^ proline has been widely used as powerful chiral catalyst for performing direct asymmetric aldol reactions. Research intensity in this direction was significantly boosted by the possibility of using simple, cheap, and small natural molecules as catalysts. Indeed, l‐proline is regarded as the simplest “enzyme” and has successfully catalyzed a lot of organic reactions with examples of aldol reactions^[^ ^220^ ^]^ and multicomponent reactions.^[^ ^58^, ^222^ ^]^ Organocatalysts like amino acids have evolved into a unique category of catalysts since they are easily accessible, environmentally friendly, and available in both of the two enantiomeric forms. However, traditional homogeneous organo‐catalysts always demonstrate unavoidable limitations, including high catalyst amount up to 30 mol% and in particular the difficulties in catalyst separation, recovery, and recycling use. Hence, innumerable attempts have been made to develop more efficient and recyclable catalysts.

**Figure 16:**
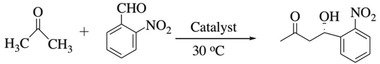
Direct aldol reaction between acetone and 2‐nitrobenzaldehyde using l‐proline/GO hybrid as chiral catalyst. Reproduced with permission.^[^ ^41^ ^]^ Copyright 2013, Elsevier.

Immobilizing small organic molecular catalysts on supports can helpfully overcome the drawbacks as aforementioned. For example, l‐proline noncovalently loaded on GO can effectively catalyze direct aldol reactions.^[^ ^41^ ^]^ The unique layered structure and the heterogeneous feature of the resulting GO‐proline hybrid catalyst favored reagents’ diffusion to the catalytic sites, thereby leading to high catalytic efficiency. High yield up to 96% and e.e. of 79% was obtained in the reaction of 2‐nitrobenzaldehyde with acetone (The reaction is presented in **Figure** **16** as a model case for direct aldol reactions). The outcome is comparable to those acquired using pristine l‐proline itself as catalyst under the equal reaction conditions. Especially noticeably, the heterogeneous hybrid catalyst can be readily recovered for recycling use. After seven repeated use, the catalyst did not show significant loss of catalytic reactivity.^[^ ^41^ ^]^ A variety of routes have been developed for attaching proline onto GO. Apart from noncovalent effects, chemical reaction can be taken to introduce proline onto GO for catalyzing aldol reaction.^[^ ^53^ ^]^ Besides, like the case with l‐proline, its derivatives, e.g., 4‐hydroxyl‐l‐proline, were also anchored on GO via covalent bonding, and the chiral hybrid proved to be an efficient, recoverable, and reusable catalyst for triggering the reactions to generate ketenes.^[^ ^58^ ^]^

The type of organic small molecules like l‐proline can be taken as artificial bio‐enzymes and efficiently catalyze asymmetric reactions. Enzymes have long been used as catalyst for performing asymmetric catalysis thanks to their high selectivity and efficiency under mild reaction conditions. Inspired by the excellent bridging between biocatalysis and photocatalysis in nature, Baeg et al. designed and constructed an artificial chiral catalyst system by a nexus incorporating photosynthetic and enzymatic processes. The catalyst was used for synthesizing enantio‐enriched alcohols from inexpensive ketones with high enantioselectivity under mild conditions.^[^ ^145^ ^]^ The photocatalytic–biocatalytic cascade approach provides new ideas for making full use of solar energy and enzymes. An excellent nexus between them would meet the demands for “green” by modern science and technologies. Intrigued by the strategy reported by Baeg et al.,^[^ ^145^ ^]^ more other judicious combinations are worthy to be devoted to more efforts, for instance, the combination of small organocatalytic with photocatalytic catalysis and even nanoreactors containing biocatalytic and photocatalytic catalyses.

l‐Proline and the derivatives are mainly used to catalyze direct aldol reaction^[^ ^41^, ^43^, ^53^ ^]^ and ketene‐forming reaction.^[^ ^58^ ^]^ Relative to proline, chiral metal complexes offer more possibilities. They can be adopted to perform a wider range of asymmetric reactions, e.g., olefin oxidation (with Mn complex),^[^ ^195^ ^]^ olefin epoxidation (Mn complex),^[^ ^196^, ^197^, ^198^, ^199^, ^200^ ^]^ sulfoxidation (Ti complex),^[^ ^202^ ^]^ hydrogenation (Rh complex,^[^ ^205^ ^]^ Ni complex,^[^ ^207^ ^]^ and Pd complex^[^ ^208^ ^]^), and ketone transfer hydrogenation (Ru complex).^[^ ^206^ ^]^ The metal complex catalysts and the corresponding asymmetric reactions are summarized in Table 6. The rich metal complex systems provide much more choices, which can be decided on according to the actual needs. Undoubtedly, chiral metal complexes acting as catalyst make more chiral compounds easily accessible.

Till now innumerable catalytically active moieties have been attached on graphene for realizing asymmetric catalysis and much more general reactions not involving chirality.^[^ ^19^, ^20^, ^21^ ^]^ The superior comprehensive properties of graphene enable it to be a powerful support for anchoring catalytic species. Hence, this research area is going to keep vigorous due to the far‐reaching implications on multiple disciplines. One potential weakness for metal complex based chiral catalysts may be the residual trace of metals, since a majority of such metals are regarded as serious contamination in pharmaceutical industries. In this regard, organocatalysts including both small organic molecules and natural biomacromolecules may be a better alternative. Notwithstanding, graphene‐based chiral catalysts are anticipated to bring some revolutionary breakthroughs for chiral‐associated areas in both academia and industries.

### 3.2 Chiral Detection

**Figure 17:**
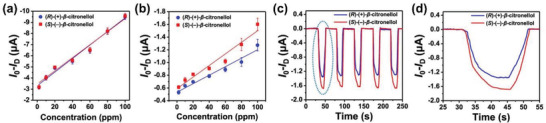
Concentration‐dependent chiral *β*‐citronellol vapor sensing signals from a) pristine graphene sensors and b) Boc‐l‐Phe‐pyrene/graphene sensors fabricated on SiO_2_/Si wafer. *I*_0_ is the baseline current. Solid lines are the interpolating calibration curves. c) Continuous enantioselective sensing responses of a Boc‐l‐Phe‐pyrene/graphene sensor device for chiral *β*‐citronellol vapor with a concentration of 100 ppm (*V*_D_ = −0.1 V; *V*_G_ = −20 V) and d) magnified plot of the dotted area in (c). Boc‐l‐Phe: Boc‐l‐phenylalanine. Reproduced with permission.^[^ ^42^ ^]^ Copyright 2018, American Chemical Society.

Chiral detection can not only determine the enantiomer in a pair of enantiomeric racemate, it also helps to diagnose diseases. For example, selectively signaling the concentration of sialic acid by circularly polarized luminescence spectroscopy may indicate the existence of cancerous cells.^[^ ^223^ ^]^ Chiral detection by combining advanced detection devices with simple chiral recognition in solution exhibits much higher potential for practical uses. Enantioselective recognition and sensing provide a fast and convenient technique to detect enantiomers. Graphene and its primary derivatives, GO and RGO, are typical 2D nanomaterials. Their excellent electrical properties offer a versatile platform for developing electrochemical sensors. If endowed with chirality, graphene derivatives provide many opportunities for the purpose of electrochemical chiral detection of chiral compounds. The chirality for creating graphene‐based chiral detectors can be derived from amino acids^[^ ^42^, ^54^, ^60^ ^]^ and cyclodextrins.^[^ ^82^, ^83^, ^84^, ^85^, ^86^, ^97^, ^98^, ^105^, ^106^, ^107^ ^]^ Kwak and co‐workers took l‐phenylalanine as chiral source and synthesized a chiral‐functionalized pyrene material (Boc‐l‐Phe‐Pyrene). It was utilized as UV–vis and fluorescent probes for enantioselective sensing. For this purpose, the chiral material was deposited as the chiral functional layer on the surface of graphene field‐effect transistors (GFETs). The as‐prepared flexible sensor device was used to recognize *β*‐citronellol vapor. Typical results are presented in **Figure** **17**.^[^ ^42^ ^]^ Figure 17a shows the change in drain current in the pristine graphene‐derived sensor device against the analyte vapor concentration. The sensor shows similar current reduction for the *β*‐citronellol enantiomer vapors. That is, the sensor is not able to enantioselectively sense chiral citronellol. As far as the chiral sensors are concerned, they show obvious enantioselective recognition ability (Figure 17b). Recycling use tests demonstrate the stability and repeatability of the chiral sensors (Figure 17c). The authors subsequently performed computation studies, and found that the enantioselective sensing effects were associated with binding interactions between the chiral analytes and chiral sensor. The binding interactions between citronellol and the sensor were chirality‐dependent, which led to the substantial differences associated with chirality. The study paves a route for creating highly sensitive and enantioselective chiral chemosensors.

Huang and coworkers prepared chiral GO using cysteine and subsequently used it to modify glass carbon electrode, which successfully recognized tartrate enantiomers.^[^ ^54^ ^]^ Fu group performed amidation reaction between the —COOH groups on GO and the —NH_2_ groups on glutamic acid to covalently bond the latter onto the former.^[^ ^60^ ^]^ The chiral hybrid was used to enantioselectively recognize 3,4‐dihydroxyphenylalanie (DOPA) racemate. Among chiral amino acids, l‐proline,^[^ ^41^, ^43^, ^53^, ^58^ ^]^ l‐phenylalanine,^[^ ^59^ ^]^ Boc‐l‐phenylalanine,^[^ ^42^ ^]^ l‐glutamic acid,^[^ ^45^, ^60^, ^61^ ^]^ d‐ and l‐cysteine,^[^ ^54^ ^]^ and l‐lysine^[^ ^62^ ^]^ are popularly used as chiral component for constructing chiral materials with graphene, GO or RGO for the target of chiral detection. Amino acids are immensely used in chirality‐related studies, for which the reasons include the wide availability, rich variety, effective cost, natural existence, and the ease in reaction with other substances in virtue of reactive amino, carboxyl, hydroxyl, and other functional groups in amino acids. In other words, amino acids offer a natural, valuable “chiral pool” for us to develop chiral materials and devices. The abundant variety and number of amino acids enable them to be ideal chiral source to satisfy various needs.

As summarized in Section 2, cyclodextrins constitute anther category chiral building blocks for chiral functionalization of graphene. Compared with amino acids, the unique cavity structure of cyclodextrins is intriguing owing to their ability to form inclusion complex with guests, especially hydrophobic compounds. This distinctive property also makes cyclodextrins appealing for chiral applications. Up to date, *α*‐CD,^[^ ^83^ ^]^ *β*‐CD,^[^ ^84^, ^85^, ^86^, ^97^, ^98^, ^100^, ^101^, ^105^, ^106^, ^107^ ^]^ and *γ*‐CD^[^ ^108^ ^]^ have been intensively employed for chiral functionalization of graphene and its derivatives. Among the three types of cyclodextrins, *β*‐CD shows the most extensive applicability as its cavity is well matched with common small chiral molecules. The chiral products derived from cyclodextrins are targeted for enantio‐differentiating objectives. For example, RGO/*γ*‐CD was used as electrode modifier for enantio‐discrimination of enantiomers.^[^ ^108^ ^]^ Similar strategies were adopted in other studies, that is, modification of electrode using chiral hybrids composed of graphene and cyclodextrin for the target of chiral recognition. For instance, RGO/*α*‐CD was used as electrode modification material for detecting chiral methionine,^[^ ^83^ ^]^ and RGO/*β*‐CD/GCE for detecting cystine enantiomers.^[^ ^84^ ^]^ Zaidi immobilized *β*‐CD on RGO and then prepared *β*‐CD/RGO/GCE sensor for recognizing phenylalanine enantiomers. The chiral sensor preferentially interacted with l‐phenylalanine over the d‐isomer. The composite sensor also showed good stability and regenerating ability, and could be further exploited for developing electrochemical enantiomer recognition sensors applicable to more chiral compounds.^[^ ^85^ ^]^

Aside from amino acid and cyclodextrin, more and more other small chiral molecules are utilized for manufacturing chiral detecting material systems.^[^ ^55^, ^121^, ^122^ ^]^ Mehdinia et al. developed a chiral sensor using betamethasone as chiral recognition element through a multilayered electrochemical deposition process, by which betamethasone, oxidized polypyrrole, and graphene sheets were deposited on the substrate.^[^ ^121^ ^]^ The deposited chiral composite film recognized the enantiomers in racemic mandelic acid. Kong et al. prepared a chiral ionic liquid and then took it as electrolyte for a one‐step synthesis of chiral graphene derivative; the chiral hybrid was employed to modify electrode for constructing electrochemical chiral sensor.^[^ ^122^ ^]^ It performed electrochemically differentiating ability toward the enantiomeric pairs of tryptophan, mandelic acid, malic acid, and tyrosine. The design and preparation strategy of chiral ionic liquids may lead to further more chiral substances of the kind, which is expected to find uses in constructing chiral materials for detection more enantiomeric compounds.

Other chiral structures offer new routes for chiral detection. For example, *R*‐mandelic acid modified calix[4]arene was used to functionalize graphene, eventually resulting in a viable electrochemical sensor toward amino propanol enantiomers.^[^ ^55^ ^]^ The studies mentioned above indicate that apart from cyclodextrin, other chiral compounds with distinctive architectures can serve as new platforms for developing chiral material systems intended for chiral detection, for instance, calixarenes^[^ ^224^, ^225^ ^]^ and crown ethers.^[^ ^226^, ^227^ ^]^ Calixarenes and crown ethers with chiral moieties can form inclusion complex with guests having commensurate size. Endowment them with chirality will lead to new types of chiral structures. Just like cyclodextrins, the chiral derivatives from calixarenes and crown ethers provide distinguished chiral building blocks for developing chiral graphene hybrids. Compared with cyclodextrins, calixarenes and crown ethers show superiority in that they can be tailored in the size of their cavity, since these synthetic macrocyclic compounds can be controlled in size at the initial design stage, according to actual requirements.^[^ ^224^, ^225^, ^226^, ^227^ ^]^ This is remarkably discernable from the natural analogues like cyclodextrins. The developed chiral materials from calixarenes and crown ethers are anticipated to show some unique functions and utilities that usual chiral materials cannot perform.

**Figure 18:**
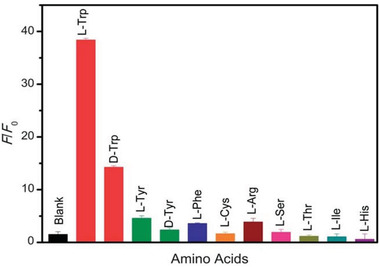
Selectivity of the amylose/graphene based chiral sensors for l‐ and d‐Trp over other amino acids. l‐Trp, d‐Trp, l‐Tyr, d‐Tyr, and l‐Phe were tested at 100 × 10^−3^ m, and other nonaromatic amino acids were tested at 10 × 10^−3^ m. The measurements were carried out in 25 × 10^−3^ m PBS (pH 6). Excitation: 400 nm; emission: 500 nm. Reproduced with permission.^[^ ^134^ ^]^ Copyright 2011, Royal Society of Chemistry.

Chiral hybrids derived from graphene and biomacromolecules also show chiral recognition ability, as the chiral small molecules do. The examined biomacromolecules include polysaccharides like amylose,^[^ ^134^ ^]^ cellulose and derivatives,^[^ ^136^ ^]^ and chitosan;^[^ ^138^, ^139^ ^]^ proteins and enzymes like human serum albumin (HAS),^[^ ^143^ ^]^ *γ*‐globulin,^[^ ^144^ ^]^ acetylcholinesterase,^[^ ^146^ ^]^ DNA;^[^ ^47^ ^]^ and even the mixture of two type of biomacromolecules, chitosan and sodium alginate.^[^ ^140^ ^]^ As a subgroup of starch, amylose is a natural linear polysaccharide consisting of d‐(+)‐glucose units linked by (1‐4)‐*α*‐glycosidic bonds. Amylose can form host–guest inclusion complex with guest substances and even with polymer chains, through hydrophobic interactions between the guests and the helical‐shaped cavity formed along amylose macromolecule chains.^[^ ^228^, ^229^ ^]^ Amylose has been substantially used as chiral selectors in chiral chromatography.^[^ ^147^, ^230^ ^]^ Compared with other biomacromolecules, e.g., proteins and DNA, amylose shows advantages due to the low cost, easy availability, convenient manipulation, etc. Qu and co‐workers developed a reusable amylose‐graphene chiral composite for sensitive and visual fluorescent sensing probe toward tryptophan enantiomers.^[^ ^134^ ^]^ Noticeably, the resulting probe exhibits highly specific selectivity for tryptophan enantiomers over the other species of the same kind, as illustrated in **Figure** **18**. The high selectivity toward tryptophan is considered being originated in the specific interaction of amylose and tryptophan.^[^ ^134^ ^]^

Herein, it is pointed out that l‐tryptophan is an essential amino acid, while d‐isomer has been taken as a precursor to synthesize neurotransmitter serotonin and anticancer drugs.^[^ ^231^, ^232^ ^]^ As a consequence, various graphene‐based chiral material systems have been created for chiral detection and separation of tryptophan enantiomers, as can be seen in Tables 1, 2, 3. Besides amylose/graphene, there have some other biomacromolecules combined with graphene or GO (RGO) for the same purpose, like chiral composite materials of consisting of chitosan/graphene,^[^ ^139^ ^]^ carboxymethyl cellulose/RGO,^[^ ^136^ ^]^ chitosan/RGO,^[^ ^139^ ^]^ chitosan/sodium/graphene/carbon nanotube,^[^ ^140^ ^]^ human serum albumin/GO,^[^ ^143^ ^]^ and DNA/RGO.^[^ ^47^ ^]^

Compared with other chiral detection and separation techniques (chromatography techniques,^[^ ^7^ ^]^ circular dichroism spectroscopy,^[^ ^233^ ^]^ chiral light,^[^ ^234^ ^]^ cavity‐enhanced polarimeters,^[^ ^235^ ^]^ microwaves, etc.^[^ ^236^ ^]^), chemosensors are attractive because of the advantages in rapid sensing ability, high sensitivity, ease of operation, small size, and the need for just a small bit of sample.^[^ ^237^ ^]^ The substantial studies already accomplished along this direction also evidently demonstrate the significant relevance of the development of chiral sensors.

### 3.3 Chiral Separation

It has been well acknowledged that enantiopure chiral species are crucially important to human beings. Their enantiomeric purity plays vital roles in biological organisms, and is considered as being one of the major means to maximize efficacy and minimize side effects of chiral pharmaceutical drugs.^[^ ^238^ ^]^ Although asymmetric catalysis has witnessed significant advancements, chiral separation is still a necessity process to acquire enantiomers with satisfying optical purity. Consequently, chiral separation has always been a critical research task with far‐reaching effects in both aspects of fundamental exploration and practical uses. In literatures, there exist diverse routes for the target of attaining enantiomers, at least the desired one enantiomer from the corresponding racemate. The widely used processes for chiral separation predominantly include chiral chromatography techniques, chiral membranes, enantioselective crystallization, and chiral adsorption. All these approaches toward chiral separation will be summarized in separate subsections below.

#### 3.3.1 Chiral Chromatography

Common chiral resolution techniques include gas and liquid chromatography and capillary electrophoresis.^[^ ^239^ ^]^ The separation mechanism lies in enantioselective interactions between enantiomer analytes and the chiral selectors. Chiral resolution generally occurs in the course of the transport of analytes through the separating materials, and can be quantified according to the difference in retention time for the two enantiomers. Nowadays, chromatography techniques have become the principle ways for chiral separation, in particular high performance liquid chromatography (HPLC) because of its relative simplicity and high efficiency. In chiral HPLC, chiral selectors are frequently immobilized on the stationary phase. Despite the powerful capability of the chiral stationary phases (CSPs) already commercialized, new CSPs are still in high requirement owing to the rapidly increasing variety and number of new chiral compounds, especially chiral drugs and agricultural chemicals. New chiral compounds also need corresponding CSPs for highly efficient resolution effects.

The CSPs in HPLC are generally composed of silica support coated by a functional chiral layer, which can be derived from chiral small molecules (e.g., cyclodextrins) or chiral biomacromolecules (e.g., cellulose, amylose, chitosan, and proteins).^[^ ^7^ ^]^ With the advancements of graphene‐associated materials and techniques, this unique type of 2D material began to be hard‐wired in CSPs used in chiral HPLC.^[^ ^127^, ^135^ ^]^ In the work published by Li et al.,^[^ ^135^ ^]^ cellulose derivatives were physically coated on RGO@SiO_2_ to prepare CSPs. With nine pairs of enantiomers as analytes (ibuprofen, trans‐stilbene oxide, 2‐phenylcyclohexanone, praziquantel, propranolol, *R*/*S*‐equol, ketoconazole, benzoin, and quinidine), the as‐fabricated chiral HPLC showed improved separation performance relative to the control column without RGO, which was attributed to the existence of additional retention interaction between RGO and analytes, such as hydrophobic effect, *π*–*π* stacking, and hydrogen bonding.

**Figure 19:**
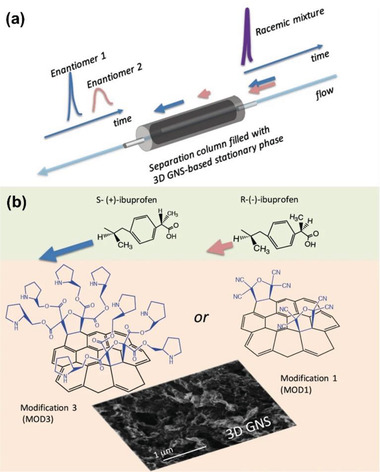
a) The principle of HPLC chiral separation: a flow of dissolved chiral drug (racemic mixture containing both enantiomers) passes a chromatographic column filled with chiral stationary phase material. Due to a difference of retention time for enantiomers of opposite symmetry, one enantiomer passes the column faster than the other, so the enantiomers are separated. b) Chromatographic separation of a chiral drug (in this case, ibuprofen in solution) with MOD3 or MOD1 3D GNS‐containing stationary phase material. The colors of backgrounds indicate the mobile phase (light olive) and the stationary phase parts (cream color) of the separation process. In the experiments, positive optical rotation angle‐enantiomers of ibuprofen and thalidomide passed through 3D GNS‐containing separation columns faster than the corresponding negative rotation angle‐enantiomers. The bottom part of the figure illustrates both chemical structure and morphology (SEM image) of mesoporous modified 3D GNS materials. GNS: graphene nanosheets. Reproduced with permission.^[^ ^127^ ^]^ Copyright 2018, Springer Nature.

Porous graphitic carbon liquid chromatography columns show high resistance against aggressive mobile phases and extreme pH of solvents/eluents, which is obviously advantageous over conventional silica‐based columns. Serov and co‐workers prepared mesoporous 3D graphene nanosheets (3D GNS), which were modified successively by tetracyanoethylene oxide (TCNEO) to form MOD1 and then (*S*)‐(+)‐pyrrolidinemethanol to form MOD3 (**Figure** **19**).^[^ ^127^ ^]^ The modified GNS was used as CSPs and demonstrated pharmaceutical‐grade chiral separation of model ibuprofen and thalidomide racemates. The chiral resolution outcome was found to be comparable to that using commercially available CSPs. Furthermore, owing to the simple covalent attachment of chiral moieties on the surface of mesoporous GNS, the as‐prepared chiral CSPs are chemically stable, and also considered being less expensive than standard silica‐based analogs.^[^ ^127^ ^]^ A schematic illustration is presented in Figure 19 to show the concept of chromatographic chiral separation using the prepared CSPs. The comprehensive performance, cost, and other properties make the newly fabricated CSPs comparable with the marketed analogues. Furthermore, the work indicates a promising direction for the development of chiral separation techniques.^[^ ^127^ ^]^

The usage of GO/*β*‐CD magnetic nanocomposites as tunable stationary phase in open‐tubular capillary electrochromatography realized a successful baseline separation for tryptophan enantiomers.^[^ ^102^ ^]^ Similarly, introducing GO and methyl‐*β*‐CD units onto the stationary phase in capillary electrochromatography improved the separation of three chiral drugs, naproxen, warfarin and pranoprofen.^[^ ^103^ ^]^ Besides cyclodextrins (*α*‐, *β*‐, *γ*‐CD), other chiral small molecules also can play the role of chiral selectors in the form of CSPs. Maltodextrin‐modified GO was coated on electrochromatographic capillary, on which chiral drugs including nefopam, amlodipine, citalopram hydrobromide, econazole, ketoconazole, and cetirizine hydrochloride were separated.^[^ ^123^ ^]^ Compared with the pristine column, the modified one significantly improved the enantiomeric separation of the six racemic drugs. Separation experiments showed that the concentration of chiral selector, concentration of GO, buffer pH, applied voltage, and concentration of buffer all exerted influence on separation process.

Capillary electrochromatography (CEC) integrates the high efficiency of capillary electrophoresis and the high selectivity of liquid chromatography phases and hence has become another powerful technique for chiral separation in the last decade.^[^ ^240^ ^]^ In CEC family, enantioselective open‐tubular CEC (OT‐CEC) provides a promising analytical separation technique due to simple column preparation, no bubble formation, and stable electroosmotic flow application. It has gained quickly increasing interest in recent years.^[^ ^241^ ^]^ Nonetheless, OT‐CEC has some shortcomings for its low‐phase ratio and sample capacity, which is due to the limited amount of stationary phase coating. The limitation restricts extending application of OT‐CEC in chromatographic separation. Fortunately, graphene offers opportunities for increasing the surface area toward coating and sample capacity. Meanwhile, biomacromolecules provide important chiral building blocks for developing chromatography techniques.

Bovine serum albumin (BSA) was conjugated with polydopamine‐GO nanocomposites and the resultant chiral hybrid was used as stationary phase in OT‐CEC.^[^ ^141^ ^]^ BSA was stably immobilized in the poly(dimethylsiloxane) (PDMS) microchannel, fabricating a protein‐type stationary phase. Further characterizations showed that the modified ones exhibited some merits like much better wettability and more stable electroosmotic mobility when compared with the native PDMS microchannels. Besides, less nonspecific adsorption was observed in the modified ones. Chiral resolution tests toward chiral tryptophan, threonine, and chiral dipeptide demonstrate that the chiral columns had high enantioselectivity. HAS and pepsin also can be selected for combining with GO, aiming at modifying affinity capillary monoliths for chiral separation uses.^[^ ^142^ ^]^ All the endeavors above provide new insights into design and construction of graphene‐based chiral selectors. Facing the quickly increasing demand for chiral compounds, the chiral composites and the resolution results based on graphene family materials will guide the follow‐up studies along the direction.

The studies focused on using graphene and derivatives after chiral functionalization may show some limitations. For the chiral graphene materials modified through weakly attaching or adsorbing chiral molecules, the chiral materials may not keep stable enough in polar organic solvents or under rigorous conditions. To overcome the deficiency, recently emerging spike of interest in graphene may provide some conceptual clues, for example, by preparing mesoporous 3D graphene nanosheets to exploit CSPs with chemical stability and cost‐effectiveness.^[^ ^127^ ^]^ Summarizing, graphene family materials offer a valuable and versatile platform for creating unprecedented chiral hybrids. In spite of the large number of studies dealing with graphene associated with chirality, there still exists a large room for further exploitation of graphene‐based chiral materials.

#### 3.3.2 Chiral Membranes

**Figure 20:**
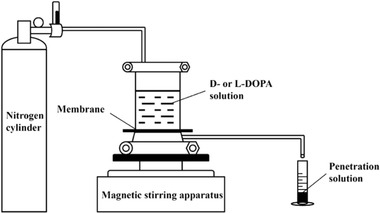
Illustration of experimental setup for chiral separation using chiral membrane. Reproduced with permission.^[^ ^45^ ^]^ Copyright 2016, Elsevier.

Chiral membranes have been explored with a relatively long history.^[^ ^242^ ^]^ Graphene and the derivatives (especially GO) provide promising candidates for developing separating materials. Prior to the popular use of graphene, chiral membranes are generally constructed by a nonselective porous support and a coating of optically active layer working as selective factor for recognizing and separating analytes. An intractable task for a most part of chiral membranes is that they are generally subjected to a trade‐off between permeability and selectivity. This academic challenge is hopefully circumvented using graphene and the derivatives. Chen and co‐workers covalently grafted l‐glutamic acid on GO and the thus‐acquired chiral hybrid was fabricated into membrane, separately using cellulose acetate and anodic aluminum oxide as support.^[^ ^45^ ^]^ The chiral membranes were examined for separating 3,4‐dihydroxy‐l‐phenylalanine racemic (l‐ and d‐DOPA), with an illustrative setup as presented in **Figure** **20**. The membranes were found to show extraordinary chiral separation properties, with 1–2 orders of magnitude higher in the flux and greater in selectivity, when compared with common chiral membranes. The results indicate that chiral‐modified graphene may provide new possibilities to simultaneously realize high flux and high selectivity. The same group further investigated the effects of host–guest interaction on chiral resolution.^[^ ^59^ ^]^ It is indicative that the nonselective interactions, although quite weak, exert large influence on the eventual separating results.

To further improve the properties of chiral GO‐derived membranes, Chen and co‐workers prepared composite chiral membranes by combining l‐glutamic acid modified GO (Glu‐GO) with a polypeptide, poly(l‐glutamic acid) (PLGA), to introduce more chiral selectors.^[^ ^46^ ^]^ More recently, the same group introduced a carboxyl‐terminated ionic liquid (IL‐COOH) as a spacer to GO, which was followed by amidation reaction using l‐glutamic acid to form the chiral hybrid (GO‐IL‐Glu).^[^ ^61^ ^]^ Hybrid chiral membranes were eventually produced and showed further enhanced separation properties. They exhibited superiority in enantioselectivity and 1–3 orders of magnitude higher in flux than traditional chiral membranes. Relative to the chiral membrane derived from l‐glutamic acid modified GO^[^ ^45^ ^]^ and Glu‐GO/PLGA,^[^ ^46^ ^]^ the three‐component membranes (derived from GO‐IL‐Glu) showed an increase of ≈40−80% in enantioselectivity and an increase of an order of magnitude in flux. The remarkable improvement relies in the ionic liquid, which not only works as spacer (favorably expanding the interlayer spacing of GO layers), but also functions as active sites to favorably improve the grafting amount of chiral selector, i.e., l‐Glu (helpfully enhancing enantioselectivity of the composite membrane). In other words, both the flux and selectivity were concurrently improved in this case.^[^ ^61^ ^]^ In a very recent work, the authors explored chiral separation mechanism through molecular dynamics simulation, using d‐alanine modified graphene as chiral membrane models and d‐ and l‐phenylalanine as chiral probes.^[^ ^243^ ^]^ This study provides guidance on development and application of chiral membranes for enantiomer separation.

#### 3.3.3 Enantioselective Crystallization

Even though chromatography techniques have become the most popular approaches for practically separating racemates, enantioselective crystallization technique is still required at present for acquiring some enantiomers.^[^ ^244^, ^245^ ^]^ Compared with asymmetric synthesis and chromatographic separation approaches, enantioselective crystallization has advantages in simple operation and cost‐efficiency. The process of crystallization is still widely utilized especially in drug mass production. Substantial progress has been made in understanding the thermodynamic and kinetic fundamentals inducing enantioselective crystallization. However, sustaining research is still important for optimizing crystallization processes.^[^ ^246^ ^]^ This technique needs the use of chiral additives as chiral inducer for triggering a certain enantiomer to preferentially crystallize or inhibiting one enantiomer from crystallization from the racemate, whereby chiral separation is realized.

Chiral graphene derivatives can serve as a special type of chiral inducers. Zhu and co‐workers reported a “grafting from” strategy for immobilizing chiral helical poly(phenyl isocyanide)s onto GO nanosheets.^[^ ^171^ ^]^ Multiple characterizations confirmed the success in grafting chiral polymer on the substrate. Chiral helical polymer chains afford GO with optical activity. The as‐prepared chiral nanocomposite was explored as chiral additive to induce enantioselective crystallization with racemic alanine as model analyte. The induced crystals have an e.e. value of 76%, displaying the potential use of the chiral nanocomposite in chiral related research areas.

**Figure 21:**
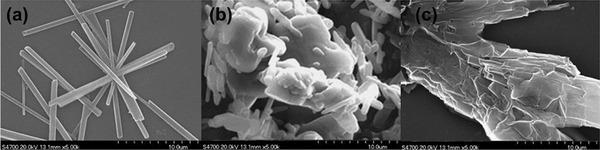
SEM images of alanine crystals obtained via enantioselective crystallization: a) using chiral GO (GO grafted with chiral helical polyacetylene); b) without additive; c) using propargyl‐GO (GO grafted with propargyl moieties). Reproduced with permission.^[^ ^180^ ^]^ Copyright 2014, American Chemical Society.

Deng group prepared chiral hybrids based on GO and chiral helical polyacetylene. A series of chiral products were fabricated, including chiral polymer chains covalently anchored on GO sheets^[^ ^181^ ^]^ and chiral polymer particles attached on GO sheets.^[^ ^180^ ^]^ Based on chiral helical polyacetylene and GO, chiral hybrid particles with diverse shapes and magnetism were also constructed.^[^ ^182^ ^]^ The chiral hybrids were used to induce enantioselective crystallization of alanine^[^ ^180^, ^181^ ^]^ and phenylalanine^[^ ^182^ ^]^ enantiomeric racemates. Taking racemic alanine as example, after inducing crystallization by chiral additives, beautiful rod‐like crystals with regular morphology were generated, which were experimentally proved to be predominantly constructed by l‐alanine (**Figure** **21**a). By contrast, no regular crystals were formed if no chiral additive was present or only achiral GO bearing polymerizable propargyl moieties was added (Figure 21b,c). Instead, only amorphous solid powder was obtained in the latter two cases, even though inducing crystallization was performed under equivalent conditions.

The findings from Deng group^[^ ^180^, ^181^, ^182^ ^]^ and Zhu and co‐workers^[^ ^171^ ^]^ definitely demonstrate the enantioselective effects of chiral helical polymer in the chiral hybrids. This type of chiral hybrids exhibits various potentials because of the presence of helical polymer chains synthesized by imitating the natural analogues like proteins and polysaccharides. In light of the fact that chiral separation is still substantially required, the primary separation processes widely used at present (HPLC and enantioselective crystallization) are going to be under continuous advancements. More efforts will be necessarily devoted to developing chiral materials as CSPs and chiral selectors in these processes. From the viewpoint of basic mechanism, the approaches involve liquid‐solid interactions and solid‐liquid equilibria. Studies focused on the aspect of exploring fundamentals may lead to new inspiration for further optimization of the chiral materials. Graphene and the derivatives offer a valuable platform for future studies. In addition, a judicious combination of diverse processes, e.g., HPLC followed by enantioselective crystallization, is anticipated to result in the desired chiral separation outcome.

#### 3.3.4 Enantioselective Adsorption

In chiral separation processes with either chromatography techniques, chiral membrane or enantioselective crystallization, the underlying driving force for the isolation of the two enantiomers from each other lies in the preferentially enantioselective interaction between the chiral selector with one enantiomer in each pair of enantiomeric racemates. In chromatographical approaches, the chiral selector means the CSPs (e.g., in HPLC), and it means the porous membranes in chiral resolution using chiral membranes; while in enantioselective crystallization, the chiral additives or chiral seeds serve as the role of chiral selectors. Furthermore, all these chiral separation processes involve liquid‐solid phase interactions. Some materials in particular those with porous 3D architectures show enantioselective adsorption ability. If the adsorption capacity and selectivity are satisfying, simple adsorption may avoid the necessity for using other tedious and high‐cost separation processes in some cases. In addition, chiral adsorption is frequently associated with academic terms such as chiral surface/interface, chirality induction, chirality transfer, etc. As a consequence, some researchers are engaged in exploring chiral adsorption from diverse viewpoints.^[^ ^247^, ^248^, ^249^ ^]^ The endeavors certainly exert positive effects on other chiral separation approaches, even on asymmetric catalysis. Additionally, in chiral adsorption processes, desorption procedure is usually carried out for acquiring enantiomeric compounds or for recycling use of adsorbent for more cycles. Accordingly, from the view point of practical operation, chiral adsorption is different from the other chiral separation techniques as described above.

**Figure 22:**
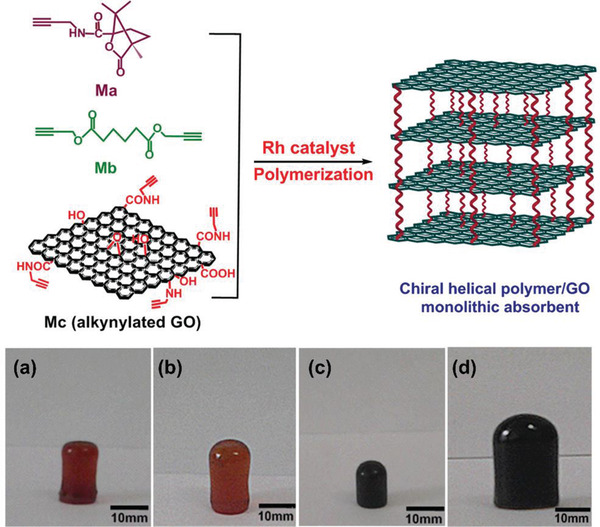
The chiral monolith constructed by GO and chiral helical polyacetylene. Ma, monomer; Mb, crosslinking agent; Mc, GO sheets grafted with polymerizable alkynyl groups. Photographs of the monolith without (Mc) a) before and b) after swelling in tetrahydrofuran for 3 weeks at room temperature; and photographs of the monolith with 15% Mc c) before and d) after swelling in tetrahydrofuran for 3 weeks at room temperature. Reproduced with permission.^[^ ^176^ ^]^ Copyright 2015, Wiley‐VCH.

Yan group grafted *β*‐CD on GO sheets, which was used for efficient resolution of asparagine enantiomers (>99% of l‐asparagine was captured in a racemic asparagine solution, while the adsorption of D‐enantiomer was not observed).^[^ ^87^ ^]^ Chiral porous materials constructed by graphene and chiral substances can be used as adsorbent for chiral adsorption. Using chiral monolithic adsorbent composed of chiral helical polyacetylene and graphene sheets (**Figure** **22**),^[^ ^176^ ^]^ enantioselective adsorption was accomplished toward phenylalanine, phenethylamine, alanine, Boc‐alanine, and leucine enantiomers. The chiral monoliths had large swelling ability; for example, the swelling degree was up to 17 times when the monolith was swelled in tetrahydrofuran. The monolith also showed remarkable enantioselectivity while adsorbing racemates. Taking phenylalanine as model analyte, the adsorption capacity toward l‐phenylalanine was over 70%, while the highest adsorption capacity was only about 25% for the d‐isomer. For the other examined racemic analytes, enantioselective adsorption was also observed. Unfortunately, the chiral monoliths were found to have some drawbacks, that is, low strength and low toughness. The reason is ascribed to the chiral helical polyacetylene chains which are generally brittle due to the rigid, conjugated polymer backbones. However, the limitations of the chiral monolith may be overcome by introducing additional flexible components.

**Figure 23:**
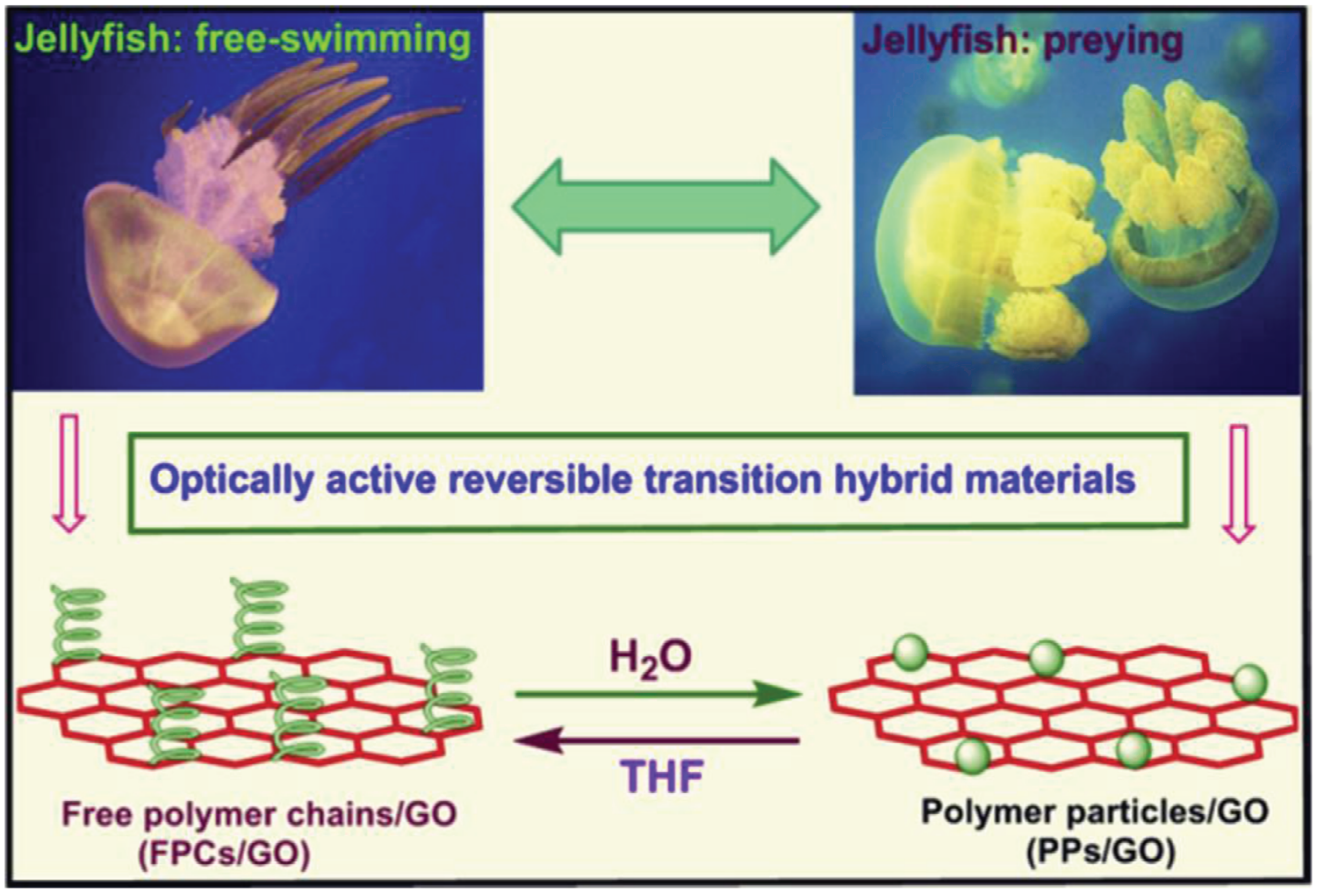
A schematic illustration for reversible transition process between free polymer chains/GO (FPCs/GO) and polymer particles/GO (PPs/GO) as response to the change in solvent mixture. Reproduced with permission.^[^ ^179^ ^]^ Copyright 2016, Elsevier.

In addition, the chiral helical polymer chains covalently grafted on GO sheets show enantioselective adsorption toward dopamine and phenylalanine racemates.^[^ ^179^ ^]^ The adsorption capacity for l‐ and d‐phenylalanine was 33% and 15%, respectively; for l‐ and d‐dopamine, adsorption capacity was determined to be 48% and 17% respectively. In this work, the authors designed and prepared chiral helical polymer‐graphene hybrid based on a biomimetic concept. More specifically, the polymer chains chemically grafted on graphene sheets undergo reversible transition between dissolution state (in good solvent, tetrahydrofuran in **Figure** **23**) and nanoparticulate state (in poor solvent of the polymer, H_2_O). The two reversibly transitional states vividly simulate jellyfish in “free‐swimming” and “preying” moments. When the polymer chains undergo the “preying” process, they behave as a chiral “predator” to “capture” one enantiomer in a pair of enantiomers. In the subsequent “free‐swimming” state, they “release” the captured guest molecules. The above processes result in chiral separation effects via the capture‐release two steps. The preparation strategy is worthy to be explored further in depth, as it provides a good example for building biomimetic architectures.

Some recently emerging materials have been utilized for constructing porous chiral materials and demonstrated tremendous potentials for developing new chiral materials. Among the new‐star materials, metal organic frameworks (MOFs) have witnessed striking progress. Like graphene, MOFs have been exploited in various research areas. Their fascinating porosity and ordered architecture enable them to be ideal building blocks for constructing chiral materials as asymmetric catalysts and enantioselective separating materials.^[^ ^48^, ^49^, ^250^ ^]^ Yu and co‐workers reported a chiral adsorbent consisting of magnetic GO and chiral MOF.^[^ ^212^ ^]^ The chiral composite was formed through encapsulating magnetic GO sheets by homochiral MOF, thus integrating the advantages of each component judiciously in one single material. Synergistic effects existed in the components, thereby enabling the chiral composite to show fast, simple, and enantioselective capture of chiral drug intermediates. Solid phase extraction tests indicate that the chiral adsorbent has remarkable selectivity toward 1,1ʹ‐bi‐2‐naphthol and 2,2ʹ’‐furoin racemates. The presence of magneticity renders the chiral adsorbent with the desirable recycling use feature. Yu et al. prepared magnetic GO‐MOF architecture, possessing both magnetic property and excellent enantioselective capture ability toward chiral drug intermediates (racemic 2,2ʹ‐furoin and benzoin) through solid phase extraction process.^[^ ^211^ ^]^ The chiral porous nanocomposites show high enantioselectivity with e.e. reaching up to 85% for 2,2ʹ‐furoin and 66% for benzoin. The capture process can be completed within 4 and 6 min, respectively. After simple washing with appropriate solvent, the chiral nanocomposite can be conveniently recovered for repeated uses. Even after six recycling use cycles, the chiral composite retains their performance without apparent loss. The work provides an interesting “prototype” in integrating 2D and porous framework for constructing advanced functional materials. As for 2D materials, besides graphene family, MXene is rising rapidly nowadays and may be used;^[^ ^250^, ^252^ ^]^ meanwhile, COF^[^ ^253^, ^254^ ^]^ can be used as porous ordered framework structures. These newly emerging materials, when combined with chirality, may open up innumerable functional materials with intriguing properties and chiral applications.

Apart from optical resolution, chiralized graphene can be utilized for other purposes. For example, Liang and co‐workers covalently grafted four types of amino acids (l‐arginine, glutamic acid, phenylalanine, and cysteine) on GO via amidation reaction, and the chiralized GO was then attached with magnetic component (Fe_3_O_4_) by chemical coprecipitation method.^[^ ^71^ ^]^ The chiral hybrids were used for magnetic separation of proteins. The authors found that the extraction capacity for proteins were dependent on the type of amino acid, pH of the solution, the absence or presence of electrolyte, extraction time and the protein concentration. Thanks to the presence of magnetic component, the chiral nanocomposite can be restored and reused with good outcome.^[^ ^71^ ^]^ Proteins are the important material basis for life and closely linked to various forms of biological processes and life activity. Furthermore, proteins are the direct manifestation of life phenomena, so the isolation, enrichment, and analysis of proteins is of great significance in biomedical areas and life science. Unfortunately, detection of proteins is still a big challenge as they are usually present in a complex sample matrix, causing intractable interference for analysis. As a consequence, sample clean‐up, separation, and enrichment become inevitable processes before further analysis of proteins. Using magnetic chiral materials to isolate proteins opens up a promising alternative for circumventing the troubles mentioned just above, for which the study from Liang and co‐workers may create a competent protocol.^[^ ^71^ ^]^

**Figure 24:**
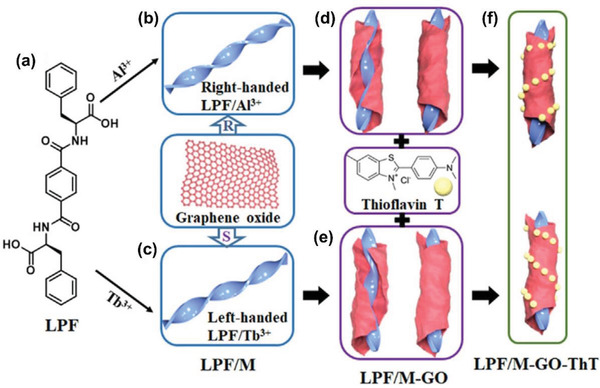
GO encapsulated LPF/M (M = Al^3+^, Th^3+^) provided hybrid materials capable of enantioselective separation and chiral molecular assembly of ThT (Thioflavin T). a) the structure of LPF; b,c) wrapping of GO onto LPF/M assemblies; d,e) LPF/M‐GO hybrids; f) chiral transcription of ThT. Reproduced with permission.^[^ ^255^ ^]^ Copyright 2020, Elsevier.

In a very recent publication, Feng et al.^[^ ^255^ ^]^ prepared chiral 1,4‐phenyldicarboxamide from phenylalanine (LPF, **Figure** **24**), which underwent self‐assembly in the presence of metal ions to form metal‐ion‐mediated l‐phenylalanine derivatives (LPF/M). GO was coated on LPF/M to form chiral hybrid hydrogels (LPF/M‐GO) through coordination interactions and hydrogen bonds between LPF/M and GO. Chiral transcription from the chiral hydrogels to thioflavin T took place through a complexation process, from which LPF/M‐GO‐ThT was generated, as illustratively shown in Figure 24. The eventually obtained chiral hybrids show enantioselective capture toward chiral analytes. Changing the metal ions provided control over enantiomer selectivity with high efficiency and reproducibility. This study gives rise to a new idea for constructing chiral materials with hierarchical structures. The strategy developed for chiral transcription is also interesting for the development of new chiral architectures.

### 3.4 Biomedical Applications

**Figure 25:**
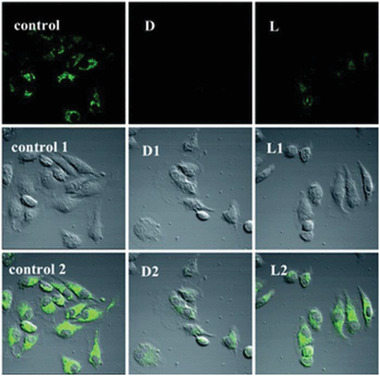
Confocal fluorescence microscopy images of HeLa cells incubated with l/d‐Phe‐F without *β*‐CD–GO (control, control 1 and control 2), l‐Phe‐F with *β*‐CD–GO (L, L1, and L2), or d‐Phe‐F with *β*‐CD–GO (D, D1, and D2) for 2–4 h at 37 °C. The images were obtained after extensive washing of cells with PBS. Bright‐field (top), dark‐field fluorescence (middle), and overlap of images of dark and bright field (bottom). l/d‐Phe‐F, derivatives from the reaction between l/d‐phenylalanine and dansyl chloride. Reproduced with permission.^[^ ^89^ ^]^ Copyright 2013, Royal Society of Chemistry.

It has been experimentally proved that biological properties of biomaterials such as cell adhesion and proliferation, protein adsorption, and antibacterial activity all exhibit chirality‐dependency.^[^ ^256^, ^257^, ^258^, ^259^, ^260^ ^]^ Biological imaging makes it possible to directly visualize the biological processes at cellular and even subcellular levels within live cells. Multifunctional biosensors have been developed and used to detect and image intracellular biomolecules.^[^ ^89^ ^]^ However, efficiently detecting intracellular chiral molecule still remains an academic challenge. To explore artificial receptors that simultaneously exhibit enantioselectivity and low toxicity in living cells, Li group utilized *β*‐CD to modify GO, from which a chiral sensor was developed. It showed excellent sensing capabilities toward d‐phenylalanine both in solution and in living cells (**Figure** **25**).^[^ ^89^ ^]^ The study creates a robust candidate for diverse biological fields, for example, intracellular imaging and intracellular tracking. GO has good biocompatibility and fluorescence‐quenching ability, and thus has captured a lot of attention in both experimental and theoretical studies dealing with its biomedical applications. Moreover, its distinguished physicochemical and structural properties allow it to be exploited as biological materials, e.g., fluorescent biosensors with intracellular imaging ability.^[^ ^32^, ^33^, ^34^ ^]^ The study from Li et al.^[^ ^89^ ^]^ opens up a new route for utilizing GO to develop biosensors. Mirabello and co‐workers prepared chiral assemblies derived from RGO‐chiral naphthalenediimides. The chiral assembled products were successfully used for prostate cancer cells bioimaging.^[^ ^124^ ^]^ The as‐developed chiral nanomaterials show prospects for biosensing and bioimaging and even other biological applications.

Disease diagnosis is another area for chiral graphene materials to perform their functions. Staden et al. prepared chiral sensors by immobilizing protoporphyrin IX, *β*‐CD, and 2,2Diphenyl‐1‐picrylhydrazyl on graphene.^[^ ^261^ ^]^ The chiral sensors were used for enantio‐analysis of glutamine in the whole blood of patients suffered from gastric cancer. The test results show that d‐glutamine can have an important role in the fast diagnosis of gastric cancer. In more detail, d‐glutamine was found only in the whole blood of the patients confirmed with gastric cancer. The study may open up a new technique for early diagnosis of gastric cancer, using chiral graphene materials as biomarker. Also notably, determination of the d‐ and l‐enantiomers’ ratio may indicate the stage of cancer, which is a much faster method than other analysis approaches. The work is expected to stimulate rapid development of chiral graphene materials applied in disease diagnosis.

Graphene‐based chiral materials demonstrate other significant application potentials in biological and biomedical area. Some chiral materials of the kind have been found to possess antimicrobial effects^[^ ^163^ ^]^ and chirality‐dependent toxicity.^[^ ^262^ ^]^ Biocompatibility of the chiral nanomaterials opens new routes for future design and developing chiral drug delivery vehicles, among other biological applications. In fact, graphene and the derivatives have been intensely exploited as drug delivery systems, in which graphene is conjugated with other components to realize the desirable biocompatibility, targeted release, enhanced drug loading ability, and so forth.^[^ ^32^, ^33^, ^34^ ^]^ Shuang et al. used *β*‐CD to modify GO and prepared chiral magnetic nanocomposite for targeted delivery and pH‐sensitive release of stereoisomeric anticancer drugs (doxorubicin hydrochloride, DOX; epirubicin hydrochloride, EPI). The study establishes an efficient nanoplatform for targeted delivery and controlled release of chiral drugs for biomedical applications.^[^ ^104^ ^]^ Enantioselective effects in chiral drug delivery has become one of the research themes in pharmaceutical community.^[^ ^263^, ^264^ ^]^ Owing to the chiral nature of the frequently used carriers and/or excipients (e.g., starch and dextrin) and the special chiral environment inside human body, chiral drugs may undergo specific enantioselective interactions with the chiral carriers and human body. Therefore, exploration delivery profile of chiral drugs becomes a significant research subject, given the percentage of chiral drugs grows rapidly at present.^[^ ^6^ ^]^ The work from Shuang et al.^[^ ^104^ ^]^ reflects that chiral graphene materials may be exploited for developing chiral carriers applicable to releasing chiral drugs, since a number of studies have shown the potential uses of graphene family materials as biomaterials.^[^ ^32^, ^33^, ^34^ ^]^ The chiral components summarized in Section 2 can be rationally coupled with graphene and the derivatives, which further improve the applicability of graphene materials. Additionally, the result in this study^[^ ^104^ ^]^ further evidences the importance of chirality in biological and physiological processes.

**Figure 26:**
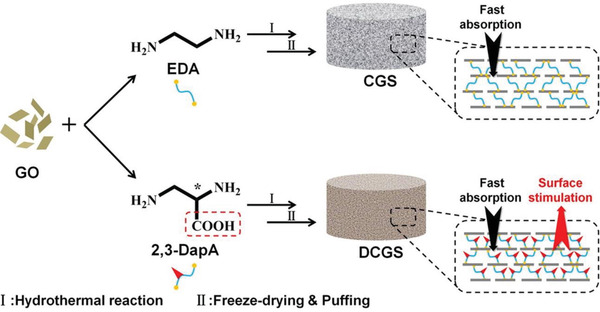
Schematic representation of the preparation route and the hemostatic mechanism.^[^ ^56^ ^]^ CGS, ethanediamine crosslinked graphene sponge; DCGS, 2,3‐diaminopropionic acid (2,3‐DapA) crosslinked graphene sponge. Reproduced with permission.^[^ ^56^ ^]^ Copyright 2016, American Chemical Society.

**Figure 27:**
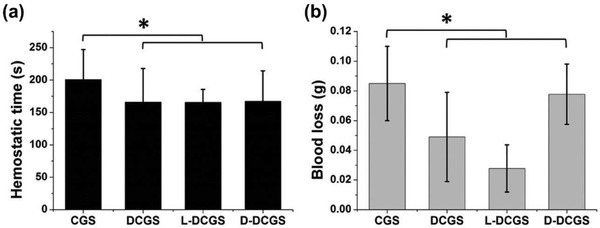
Data from the rat‐tail amputation model among the CGS, DCGS, l‐DCGS, and d‐DCGS. a) Hemostasis time. b) Blood loss. Data values corresponded to mean ±SD, *n* = 6. * means *p* < 0.05 and represents a significant difference compared with the CGS. l‐DCGS and d‐DCGS, l‐ and d‐2,3‐diaminopropionic acid (l‐ and d‐2,3‐DapA) crosslinked graphene sponge, respectively. Reproduced with permission.^[^ ^56^ ^]^ Copyright 2016, American Chemical Society.

Aside from the use for drug delivery systems, chiral graphene composites were also investigated as hemostasis.^[^ ^56^ ^]^ Wang et al. used 2,3‐diaminopropionic acid (DapA) to crosslink graphene sponge (DCGS) for hemostasis treatment (**Figure** **26**). Relative to a reported ethanediamine crosslinked graphene sponge (CGS), DCGS has carboxyl groups and not only quickly absorb plasma, but also stimulate erythrocytes and platelets to change their normal form and structure at the interface. The effects largely affect a cell's metabolism and biofunction, further promoting blood coagulation. Whole blood clotting and rat‐tail amputation tests show that the hemostatic efficiency of DCGS was significantly improved compared with that of CGS control sample, as illustrated in **Figure** **27**. This improvement is considered being attributed to the increased oxidation degree and the negative charge density. The study indicates that on the premise of keeping fast absorption, enhancing the cell/graphene interface interaction may be an effective way to improve hemostatic efficiency. In addition, the chiral effect of DapA was demonstrated on blood cell adhesion rather than hemostatic performance. The work not only directs a new avenue for developing graphene‐based hemostatic materials, but also presents new insights into chiral materials for use in bleeding control. More efforts devoted to elucidating hemostatic behaviors’ correlation with chirality may shed light on chiral biomaterials.

### 3.5 Other Applications

Graphene and the derivatives exhibit many intriguing properties, and on the other hand, studies dealing with chirality has expanded to multitude areas. Undoubtedly, the composite materials combining them together possess substantial application potentials. Besides the major uses of chiral graphene materials as summarized above, there are also some other applications with great promise. Qing and co‐workers grafted cysteine as a model of chiral species on GO to elucidate whether surface chirality has remarkable effects on protein misfolding and forming amyloid aggregates.^[^ ^44^ ^]^ It has been known that protein amyloidosis is closely associated with many neurodegenerative diseases like Alzheimer's and Parkinson's diseases.^[^ ^265^ ^]^ On the other hand, it becomes gradually clear that molecular surface in particular biological membranes play crucial roles in the process of protein amyloidosis.^[^ ^266^ ^]^ In theory, chirality of the biological substances’ surface may be directly related with protein misfolding process. To justify this consideration, Qing and co‐workers^[^ ^56^ ^]^ accomplished the study and found that *R*‐cysteine modified GO surface suppresses the adsorption, nucleation, and fiber elongation processes of the examined protein, and thus drastically inhibited amyloid fibril formation on the surface; on the contrary, *S*‐cysteine modified GO promotes these processes. Furthermore, surface chirality also markedly influences the conformational transition of the protein from *α*‐helix to *β*‐sheet. The study gives rise to novel insights into better understanding of the amyloidosis process occurring to proteins on surface from a biomimetic perspective. The material prepared in this study also offers an importance platform for investigating in depth biological effects that play vital roles in keeping human health. It has been unambiguously demonstrated that chirality of biomaterials has substantial effects on adhesion, enrichment, and migration of cells and adsorption of proteins.^[^ ^7^, ^147^, ^242^, ^245^ ^]^ Accordingly, a new prerequisite seems to emerge for the new generation biomaterials; that is, their chirality should be taken into consideration from the starting point of design.

Chiral graphene materials may further find actual applications in photoelectric area. Even though the efforts along this research direction are still scarce yet, the application potentials of such chiral materials have been demonstrated. For example, Zhu et al. developed high‐performance thermal management materials for dissipating excessive heat both in plane and through plane, which is of large interest to maintain efficient operation and prolong electronic devices’ life. For this purpose, the authors constructed a graphene‐derived composite film through conjugation with chiral cellulose nanocrystals (CNCs).^[^ ^267^ ^]^ Chiral smectic liquid crystal structures were formed in the composite film. The helical arranged nanorods of carbonized CNCs played the role of in‐plane and through‐plane connection, thereby bridging graphene layers together. The chiral helical architecture enabled the composite film to exhibit extraordinary thermal conductivity in both in‐plane and through‐plane (1820.4 and 4.596 W M^−1^ K^−1^, respectively). Moreover, the film showed high heat transportation efficiency, excellent mechanical strength, and electrical conductivity. The created chiral composite film has potential for developing high‐performance thermal management films. In particular worthy to be mentioned is the chiral helical architecture formed by rod‐like CNCs. This distinctive construction enables the chiral composite material to show the appealing properties and huge application potentials yet to be explored systemically.

Nowadays, some architectures with nano‐ and microdimensional and unique profiles are gathering quickly increased interest. As a typical 2D material, graphene and the analogs have demonstrated numerous application potentials, as summarized in this overview paper. If the 2D material is rationally combined with a 1D material, e.g., CNCs, fascinating findings are high anticipated from the resulting composite. Some endeavors have been made under this topic. The judicious incorporation of graphene and CNCs may provide advanced functional materials with promising applications not only as thermal management^[^ ^267^ ^]^ but also as optical devices.^[^ ^137^, ^268^ ^]^ Zhu et al. reported a chiral smectic architecture prepared through self‐assembly of graphene oxide sheets and CNCs nanorods. The material with chiral smectic possess potential applications as optical metamaterials in optical modulation and mechanochromic sensing devices.^[^ ^137^ ^]^ As far as graphene‐based chiral liquid crystal materials are concerned, Feng and co‐workers recently published an overview paper, which can be referred to for more information.^[^ ^268^ ^]^ More detailed optical applications of chiral liquid crystal materials derived from graphene are reviewed therein. Graphene‐derived hierarchical and chiral materials have demonstrated promising application prospects in the currently active research area of energy storage, as reported by Tao and co‐workers^[^ ^51^ ^]^ Despite the limited studies at present, the unique structures and properties of graphene derivatives and the significant importance of chiral architectures will facilitate the chiral composite materials to find a wide spectrum of actual applications. The new‐generation materials might exert far‐reaching effects in modern science, technology, and human daily life.

## 4 Newly Emerging Chiral Graphene Hybrid Materials

Graphene based chiral hybrid materials are under rapid progress currently. In spite of the rich variety and myriad of such chiral materials already created so far, more new chiral hybrids of the kind are still under active development. Graphene constructed chiral materials with more complex architectures and more fascinating functions are continuously emerging. More other components are introduced in graphene material systems to fabricate chiral hybrids with distinctive properties and applications. The emerging chiral hybrids currently under intensive exploration have demonstrated their superiority in new structures, properties, and functions.

### 4.1 Graphene Quantum Dots Based Chiral Graphenes

Graphene quantum dots (GQDs) refer to graphene sheets generally smaller than 10 nm. They possess similar fundamental properties to graphene. Nonetheless, compared with usual graphene sheets, GQDs have unique quantum confinement and edge effect (much more oxygen‐containing functional groups on edges) and thus have some fascinating properties that general 2D graphene sheets do not have.^[^ ^269^, ^270^ ^]^ Accordingly, GQDs have drawn rapidly growing attention from various research communities. In addition, their excellent photostability, biocompatibility, and small dimension enable them to exhibit tremendous application prospects in particular in biomedical applications,^[^ ^271^ ^]^ sensing,^[^ ^272^ ^]^ batteries,^[^ ^273^ ^]^ LEDs,^[^ ^274^, ^275^, ^276^ ^]^ and as surfactant for polymerization.^[^ ^270^ ^]^ Particularly, the properties of small size, large specific surface area, and abundant edge sites endow GQDs with superiority for electrochemical sensing.^[^ ^269^, ^272^ ^]^ Consequently, some groups take GQDs for developing chiral electrochemical sensors.^[^ ^277^, ^278^ ^]^ Unfortunately, pristine GQDs do not have chiral structure. To afford chirality to them, so far primarily two strategies have been developed, as to be analyzed below. The chiral materials derived from GQDs are summarized in **Table** **7**.

**Table 7:** Overview of chiral components utilized for developing chiral graphene quantum dots and their applications

Chiral component	Application^a)^	Ref.
*β*‐CD	Electrochemical recognition (tryptophan)	^[^ ^277^ ^]^
*β*‐CD	Electrochemical sensor (tyrosine)	^[^ ^278^ ^]^
Tartaric acid	Electrochemical chiral sensing (tryptophan)	^[^ ^279^ ^]^
Cysteine	Chirality‐dependent toxicity and biological activity	^[^ ^262^ ^]^
2‐Phenyl‐1‐propanol	Chirality transfer to other matter	^[^ ^280^ ^]^
*β*‐CD/cellulose	Chiral stationary phase (ten pairs of enantiomers)	^[^ ^281^ ^]^
Chitosan	Chiral recognition (tryptophan)	^[^ ^282^ ^]^
chitosan	Electrochemical chiral recognition (tryptophan)	^[^ ^283^ ^]^
Bovine serum albumin	Chiral recognition (tryptophan)	^[^ ^284^ ^]^

^a)^ The corresponding analytes for chiral recognition are presented in the brackets.

**Figure 28:**
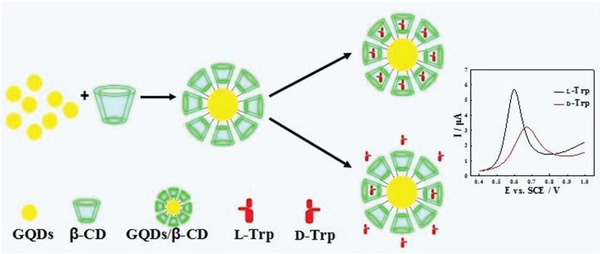
Schematic illustrating the preparation of GQDs/*β*‐CD chiral nanocomposites and the recognition of Trp (tryptophan) isomers by the nanocomposites. Reproduced with permission.^[^ ^277^ ^]^ Copyright 2015, Elsevier.

Two strategies have been developed for constructing chiral GQDs. The first strategy is “bottom‐up” method, well exemplified by the studies from Martín et al.,^[^ ^38^ ^]^ Maçoas and co‐workers,^[^ ^39^ ^]^ and Segawa and co‐workers ^[^ ^40^ ^]^The second one can be denoted as “postchiralization” method, typically exemplified by the study from Haino et al.^[^ ^285^ ^]^ In the two approaches, the second one has witnessed rapid advancements due to the relatively easy introduction of chiral moieties onto GQDs either through noncovalent interactions or covalent bonds.^[^ ^277^, ^278^, ^279^ ^]^ Kong et al. reported a method to combine cyclodextrins with GQDs by noncovalent interactions, as presented in **Figure** **28**.^[^ ^277^ ^]^ In this study, GQDs were prepared by pyrolysis of citric acid. They were then incorporated with *β*‐CD via hydrogen bonds occurring between the oxygen‐containing groups on GQDs and the hydroxyl groups on *β*‐CD. The as‐obtained chiral hybrids of GQDs/*β*‐CD were negatively charged due to the ionization of carboxyl groups on GQDs. The chiral hybrid was subsequently electrodeposited onto GCE for implementing the objective of electrochemically recognition toward tryptophan (Trp) enantiomers. The recognition results are displayed in Figure 28, showing that the inclusion complex of GQDs/*β*‐CD/l‐Trp generates a significantly higher peak current than GQDs/*β*‐CD/d‐Trp. The peak current ratio of the former to the latter one is calculated to be 2.1. The results indicate that *β*‐CD preferably complexes with l‐Trp enantiomer, owing to the favorability of hydrogen bonds formed between the secondary hydroxyl groups on *β*‐CD rims and the amino group of l‐Trp, when the indole group of Trp enantiomers inserts into the cavity of *β*‐CD units.^[^ ^277^ ^]^

In other reports, Kong et al. prepared GQDs/tartaric acid chiral hybrids for electrochemical recognition of Trp enantiomers.^[^ ^279^ ^]^ Zhao et al. prepared GQDs/*β*‐CD chiral hybrids for discrimination of tyrosine enantiomers.^[^ ^278^ ^]^ The aforementioned studies provide convenient techniques for fast detection of chiral enantiomers. The above chiral hybrids were constructed by GQDs/chiral small molecules via noncovalent interactions. In some other studies,^[^ ^262^, ^280^, ^281^ ^]^ the two major components, that is, GQDs and chiral substances, were connected through covalent bonds. Herranz and co‐workers prepared chiral GQDs successively by acidic exfoliation and oxidation of graphite, followed by dialysis and esterification with enantiomerically pure (*R*)‐ or (*S*)‐phenyl‐1‐propanol. The chiral GQDs formed supramolecular aggregates with pyrene molecules. More interestingly, chirality transfer occurred from the chiral GQDs to pyrene units.^[^ ^280^ ^]^ In the study from Violi and co‐workers,^[^ ^262^ ^]^ GQDs were prepared and then covalently attached with l‐ or d‐cysteine using the carboxylic groups on GQDs and the amino group on cysteine to undergo amidation reaction. With covalently grafted cysteine moieties, the edges of the chiralized GQDs became crowded and then helical buckling occurred. The helicity of the buckled structures is associated with the chirality of the preintroduced cysteine. UV–vis absorption spectrum of the pristine GQDs revels a peak around 228 nm. For the chiral GQDs, the UV–vis absorption peaks shifted to 265 nm. CD spectra show that the l‐ and d‐cysteine functionalized GQDs have CD effects with opposite signs. The chiroptical asymmetry parameter (*g*‐factor) of chiral GQDs at the range of 250−265 nm is equal to 1 × 10^−4^, comparable to that of many chiral organic compounds. Further experimental and modeling data indicate that 3D twisting occurred to the graphene layers. In vitro evaluation tests of the GQDs were accomplished against liver cells to explore their biocompatibility. It is demonstrated that the GQDs have low cytotoxicity and differentiation of cytotoxicity between GQDs stereoisomers. Biocompatibility of the chiral GQDs opens up new routes for developing chiral drug delivery vehicles and more selective phototherapies; the twisted electronic states may lead to polarization‐based optoelectronic devices and new types of chiral catalysts.

GQDs combined individually with *β*‐CD and cellulose can enhance the enantioseparation performance of *β*‐CD and cellulose, referring to the work from Qiu and co‐workers^[^ ^281^ ^]^ According to the experimental and molecular simulation data, GQDs provide extra interactions such as hydrophobic, hydrogen bonding, and *π*–*π* interactions while the chiral selectors (*β*‐CD units and cellulose macromolecular chains) enantioselectively interact with analyte enantiomers. The synergistic effects help to enhance the chiral recognition ability of the GQDs‐modified chiral hybrids. In this study, cellulose was combined with GQDs via noncovalent interactions. Besides cellulose, GQDs also can be combined with other biomacromolecules (see below), resulting in advanced functional chiral materials. Striking progress has been made under the research direction.

GQDs were combined with chitosan, from which a chiral composite film was fabricated. The film was prepared by successive eletrodeposition of GQDs and chitosan on the surface of a GCE.^[^ ^282^ ^]^ Owing to the strong interaction between GQDs and chitosan, the resulting chiral composite film was regular and uniform, and then applied to electrochemical chiral recognition of Trp enantiomers. In the chiral discerning process, chitosan provided a chiral microenvironment, while GQDs further amplified the electrochemical signals and thereby improved the recognition efficiency. In other words, the desired synergistic effects occurred between chitosan and GQDs, offering the chiral recognition toward Trp enantiomers. The work extends the applications of GQDs to electrochemically chiral sensors. Electrochemical recognition toward Trp enantiomers was also realized using self‐assembled architectures consisting of four components: GQDs, diphenylalanine, chitosan, and CTAB (cetyltrimethylammonium bromide).^[^ ^283^ ^]^ The assembled products showed enantiomerically differentiating ability. GQDs can also be combined with biomacromolecules through covalent bonds. Kong et al. functionalized bovine serum albumin (BSA) with GQDs.^[^ ^284^ ^]^ The obtained chiral hybrid served as a sensing platform for chiral recognition of Trp enantiomers. To form the covalently bonding connection, amidation reaction between BSA and GQDs was implemented. The resulting chiral sensor exhibited good biomolecular homochirality in recognizing Trp isomers, in which the chiral hybrid showed higher affinity toward l‐Trp than the opposite d‐isomer.

The above studies focused on combination of GQDs and chiral small biomolecules and biomacromolecules in one single material clearly demonstrate that GQDs and the chiral components provide their individual advantages. Synergistic effects can be anticipated from the two building blocks. In view of the good biocompatibility of both the two components, the chiral hybrids have promising potential applications such as biosensors, nanomedicines, and nanocarriers for delivering drugs. The materials of the kind are under intensive exploitation currently, but have shown bright prospects acting as a new generation of biomaterials. Aside from the biocompatibility, the chirality also enables them to be highly qualified for bio‐applications. Such chiral biomaterials are anticipated to be further developed as a new generation of biomaterials, since the routine counterparts lack the characteristic feature of chirality.

### 4.2 Graphene Materials with Constructional Chirality

A majority of graphene‐based chiral materials are constructed by attachment of external chiral components onto graphene sheets, as demonstrated in the above sections. It means that the chirality of such chiral materials is stemmed from the attached structures bearing configurational chirality (small chiral molecules), conformational chirality (helical polymers) or a hierarchal chiral structure (chiral liquid crystal structures). Obviously discernable from these studies, chirality of some graphene and the derivative materials comes from constructional chirality. At this point, it is important to remark that to distinguish from configurational chirality, conformational chirality, and hierarchal chirality as mentioned above, constructional chirality refers to the chiral graphene materials in which the chirality is originated in factors rather than chiral molecules, biomacromolecules, polymers, chiral metal complexes, and chiral liquid crystals. Instead, constructional chirality is closely associated with asymmetric stacking of graphene sheets, or wrinkles with asymmetric architectures. In brief, constructional chirality is not derived from chiral (macro)molecules and other external elements, but from graphene sheets themselves. Compared with the ones with chiral (macro)molecules as chiral source, graphene materials possessing constructional chirality exhibit distinct characteristics in both structure and properties, and probably also in their functions and applications.

**Figure 29:**
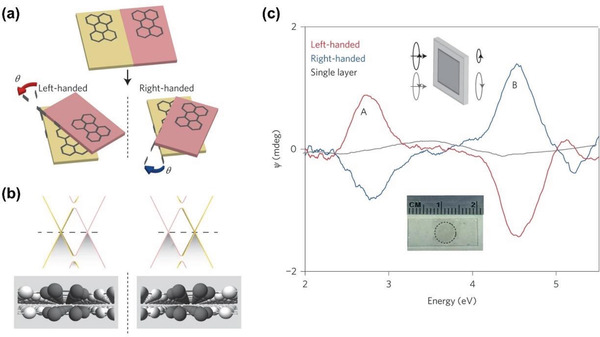
Chiral atomically thin films produced by chiral stacking. a) Schematic of the chiral stacking process for generating left‐ and right‐handed twisted bilayer graphene films connected by a mirror plane (vertical line). b) Electronic band structures (top images) and cross‐sectional schematics with spiral atomic arrangements (bottom images) of left‐ and right‐handed twisted bilayer graphene films. c) Ellipticity (*Ψ*) spectra or circular dichroism (CD) spectra, measured from a pair of chiral twisted bilayer graphene films with *θ* = 16.5° (red: left‐handed, blue: right‐handed) and single‐layer graphene (gray). The CD spectra of left‐ and right‐handed twisted bilayer graphene each show two strong peaks, denoted Peak A and B. Lower inset: Photograph of a twisted bilayer graphene film (circled area; 5 mm in diameter) on a fused silica substrate. Upper inset: Schematic for *Ψ* measurements, where twisted bilayer graphene absorbs left‐ and right‐handed circularly polarized light differently. Reproduced with permission.^[^ ^286^ ^]^ Copyright 2016, Springer Nature.

Park et al. prepared chiral atomically thins films through a chiral stacking approach,^[^ ^286^ ^]^ as illustratively shown in **Figure** **29**. The preparation approach involved precisely controlling interlayer structures (*θ* and rotational polarity). The stacking procedure included two steps. In the first step, a single‐layer graphene film was grown with a uniform crystalline orientation over the entire film; In the second step, the film was cut into pieces which were subsequently stacked layer‐by‐layer with a controlled *θ* angle on the basis of the known crystalline orientation while rotating anticlockwise or clockwise to form left‐ or right‐handed films connected by a mirror plane (vertical dashed line). Since the graphene sheets were positioned layer‐by‐layer with precise control of the interlayer rotation and polarity, the eventually resulting film exhibited tunable chiral properties correlated with the packing of graphene sheets. The chirality of the assembled films was confirmed by CD spectra measurement (Figure 29c).^[^ ^286^ ^]^ The fabrication methodology and the tunable chiral properties may be extended from graphene to other 2D layered materials (e.g., MXene^[^ ^251^, ^252^ ^]^) to form chiral atomically thin film devices. It also opens up new approaches for realization and adjustment of chiral properties showing diverse electrical and optical characteristics. The concept of chiral stacking approach built in this work allows the investigation of the coupling between structural chirality and the spin and valley degrees of freedom. In addition, the chiral stacking approach and the fundamental exploration of the materials’ chirality dependent properties may promote the development of circuits integrating multiple functions together, which may exhibit advanced electrical, optoelectronic and chemical sensing performances.

Especially noticeably, the chirality of the graphene films^[^ ^286^ ^]^ was realized via chiral stacking rather than using additional chiral source as done in general endeavors. The study presents a fundamental understanding of the construction of materials’ chirality depending on structural asymmetry instead of (macro)molecular configurational or conformational asymmetry. Together with other chiral materials whose chirality is alike derived from stereo‐constructional asymmetry rather than molecular level asymmetry,^[^ ^287^, ^288^ ^]^ the chiral graphene materials created in the work^[^ ^286^ ^]^ demonstrate that chiral architectures consisting of left‐ and right‐handed counterparts linked by mirror symmetry are useful for developing advanced electrical and optoelectronic applications such as polarization optics and spintronics.^[^ ^289^ ^]^

In virtue of external physical forces, graphene‐based materials can be awarded with constructional chirality, making the use of additional chiral molecular moieties unnecessary. Zhang et al. reported an interesting approach for fabricating assembled chiral nanoarchitectures (nanofibers, nanorings, etc.) in vigorously stirred polymer solutions. Such chiral nanoarchitectures can be derived not only from 2D graphene nanosheets, but also from other nanomaterials like nanowires and nanotubes.^[^ ^287^ ^]^ The underlying driving forces for realizing the chiral self‐assemblies are vigorous stirring and the presence of a polymer. It has ever been reported that stirring can induce chirality in initially achiral molecules and even induce circularly polarized luminescence from achiral materials.^[^ ^290^, ^291^, ^292^ ^]^ These findings indicate that physical stirring not only induces chirality at (macro)molecular level, but also at much higher levels. The investigation opens up a facile and universal strategy for self‐assembly of various low‐dimensional structures (nanoparticles, 1D and 2D nanostructures) for fabricating hierarchical chiral nanoarchitectures, like chiral nanofibers and nanorings and even more complicated architectures. Apart from carbonaceous materials, more other materials with diverse compositions and dimensionalities such as magnetic nanoparticles, metal nanowires, semiconductor quantum dots and so on can be assembled into chiral multiscaled materials.

Wang and co‐workers developed a process for inducing chirality in GO based hybrid materials. GO and humic acid (HA) assembled into sandwich‐like complex which further assembled into twisted, long‐range‐ordered nanostructures via noncovalent interactions like electrostatic repulsive effects, *π*–*π* stacking, and hydrogen bonding between GO sheets and HA. Rippled GO‐HA layers were generated and further assembled to form macroscopic chirality, due to interlayer and interdomain distortion which led to a twisted conformation of the C=C double bonds.^[^ ^288^ ^]^ Briefly, in the assembly process, ripples were formed in single GO‐HA layers due to noncovalent interactions. Such wrinkled layers further assembled into higher level structures having twisted domains. The twisting structures rendered the ultimately generated materials with constructional chirality. There have been various strategies for producing polymeric materials with wrinkles at diverse scales.^[^ ^293^ ^]^ Polymeric materials may be wrinkled in bulk or have wrinkled surface through self‐assembly,^[^ ^294^, ^295^ ^]^ interpenetrating polymer network,^[^ ^296^ ^]^ or under mechanical strain.^[^ ^297^ ^]^ Once coupled with chirality in the course of producing the wrinkles, a great number of hierarchically structured materials may be developed, which are expected to show macroscopic chirality because of their asymmetric hierarchical architectures.

The studies above^[^ ^286^, ^287^, ^288^ ^]^ have a common point in that the chirality (more specifically, the macroscopic chirality) was induced by special nanostructures (twisted stacking, wrinkles, etc.) or twisting force (stirring) rather than by deliberately adding external chiral inducers. The preparation strategies are inspiring, might stimulating more new ideas for constructing chiral hybrids starting from graphene or GO. Theoretical studies indicate that graphene and the derivative sheets could have weak electronic chirality because of the existence of geometric distortion on the carbon hexagonal planar structure.^[^ ^298^ ^]^ Now it has been further demonstrated that rippled domains in 2D graphene sheets can be induced by external forces, thereby forming highly anisotropic geometry and generating constructional chirality at relatively macroscaled levels.^[^ ^286^, ^288^ ^]^ Herein, it may be interesting to consider that if an additional chiral component is present in the process of producing wrinkles or twisted architecture, what would be the finally formed material? How about its micro‐ and macroscaled chirality? The as‐prepared materials, even though still at conceptual design stage, seem to be so highly fascinating that experimental exploration may be undertaken soon, from which unexpected findings are highly anticipated.

### 4.3 Multifunctional Chiral Graphene Hybrid Materials

Some special components are added in hybrids constructed from graphene and chiral moieties to afford distinctive properties that cannot be achieved in routine chiral graphene materials. One example is to combine magnetic ingredient (Fe_3_O_4_ nanoparticles) into the chiral hybrid derived from graphene, which endows the chiral hybrid with magneticity and further the ready restoration and recycling use thereof.^[^ ^299^ ^]^ The resulting magnetic chiral sensing system can specifically recognize histidine enantiomers. In another work, chitosan‐based molecularly imprinted polymers (MIPs)^305^ were integrated with RGO to subsequently modify glassy carbon electrode (GCE).^306^ Herein, *S*‐propranolol was taken as the template for producing MIP. The chiral sensors fabricated in this study demonstrated electrochemically enantioselective recognition toward chiral propranolol drugs (*S*‐ and *R*‐propranolol in the presence of interference substances: metoprolol, lidocaine, atenolol, carvedilol, and alprenolol). The endeavors^[^ ^299^, ^301^ ^]^ provide some stimulations for further developing chiral hybrids based on graphene and even other organic and inorganic materials. MIPs, which have “lock‐key” features,^[^ ^300^ ^]^ when reasonably incorporated with graphene chiral materials, the as‐prepared chiral composite materials are expected to show a wide range of advantages, including high porosity, high selectivity, and optical activity as well. The high selectivity may enable the chiral materials to be qualified for actual uses and at the same time to be advantageous over the analogues. Such chiral materials bearing MIPs might find broad practical uses in sensing, separation, adsorption, catalysis, drug delivery, among other applications.

**Figure 30:**
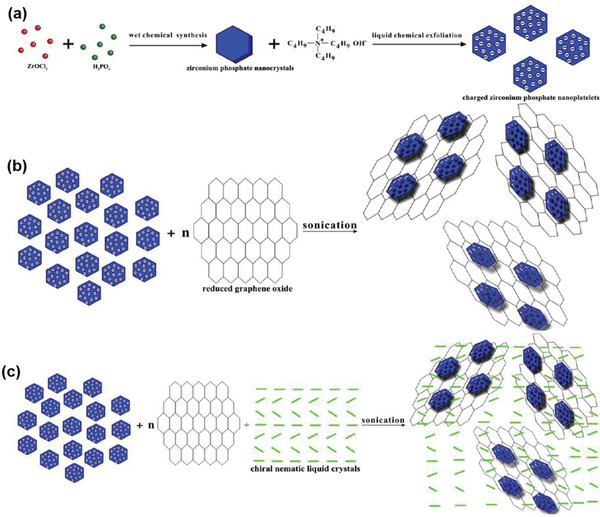
a) Schematic illustration of synthesis of 2D CZ nanoplatelets, debundling RGO in b) liquid phase, and c) dispersing and assembling RGO in CNLCs by CZ c). CZ, charged zirconium phosphate; CNLCs: chiral nematic liquid crystals. Reproduced with permission.^[^ ^302^ ^]^ Copyright 2017, Elsevier.

Some fascinating structures have been realized starting from graphene and the derivatives. Cheng et al. prepared RGO composite possessing chiral helical configuration.^[^ ^302^ ^]^ As shown in **Figure** **30**, the authors first prepared a surfactant (2D charged zirconium phosphate, CZ) and used it to disperse RGO in liquid phase. Then the mixture was further dispersed in an ordered soft matter (chiral nematic liquid crystals, CNLCs). Eventually the ternary CZ‐RGO‐CNLC composite was formed and possessed chiral helical structures. In this work, the dense negative charges on CZ nanoplates were beneficial to induce electrostatic repulsion onto RGO. Moreover, the 2D nanostructure of CZ could render the favorable steric hindrance between RGO layers. As a result, the unique structure of CZ effectively inhibited the recombination of RGO sheets in the dispersion mixtures. With the assistance of CZ, RGO sheets further assemble din CNLCs to form an ordered architecture. The eventually resulting composites combined the superiority of both RGO and CNLCs and might work as an ordered soft matter, providing opportunities for fabricating multifunctional and smart materials and devices.

In the study from Zhu et al.,^[^ ^137^ ^]^ 2D GO and 1D CNCs self‐assembled to form a chiral smectic structure. It was found that the formation of the chiral structure was strictly dependent on the ratio of the 2D GO to the 1D CNCs and their concentration. The chiral material opens up a new way for developing mesoscopic materials with tunable physical properties potentially as optical metamaterials that might be used in the construction of optical modulation and mechanochromic sensors. Some other 1D structures (rod‐like polymer chains, semiconductor and metal rods, etc.) and 2D materials (MXene and inorganic material nanoplatelets) provide a variety of candidates for developing chiral architectures of the kind. Their individual electronic, optical, mechanical properties and so forth may render the chiral composite materials with substantial uses.

Chiral supramolecular architectures have witnessed substantial progress in recent years. The endeavors can favorably further our understanding of nature's ability to create functional materials that possess hierarchical structure over a wide range of size scale. Inspired by the hierarchical structures in nature, biomimetic architectures can be developed.^[^ ^303^, ^304^, ^305^ ^]^ Nowadays the quest for effectively controlling and predicting the chirality of supramolecular artificial materials still keeps a highly active research topic, attracting the attention of scientists from diverse disciplines. The developed human‐made chiral building blocks and chiral supramolecular assemblies have proved to be of significant and wide applicability to a range of uses in detection and separation of chiral compounds and more other areas related to chirality.^[^ ^306^ ^]^ Based on graphene and helical silica nanotubes, Jung and co‐workers developed a type of chiral hybrid material through electrostatic assembly.^[^ ^307^ ^]^ The chiral hybrid integrates the electrochemical properties of graphene and enantioselective separation ability of helical silica. The authors realized the encapsulation of helical silica nanotubes with RGO sheets, providing a chiral hybrid material being capable of chiral transcription of organic substances attached on the surface. Thereby, the study demonstrates that chiral transcription can be implemented with the assistance of graphene sheet surface.^[^ ^307^ ^]^ Chiral transcription was also realized using GO sheets, as reported recently.^[^ ^255^ ^]^ The resulting chiral hybrids may be applicable to other chiral applications like chiral sensing and recognition, besides chiral separation. The studies above open up a new way for creating novel chiral functional materials by taking advantage of graphene and other components with distinctive architectures and properties.

## 5 Challenges and Perspectives

### 5.1 Challenges

It has been clearly demonstrated that numerous unprecedented chiral materials will be generated once graphene family materials are reasonably integrated with chirality feature. Indeed, the potential variety and number of such chiral materials are tremendous. Referring to the literatures and based on the studies from our owns, some challenges still exist at present, in spite of the striking advancements made in the construction and exploration of graphene‐based chiral materials. The challenges are stated as follows.

1) Characterization of chirality. How to quantitatively determine the chiral structure and the corresponding optical activity of the chiral hybrids still remains a big academic challenge, since nearly all the hybrids are solid. Therefore, unlike the samples in solution state, in which the characterizations including CD spectra, optical activity and so on can be performed quantitatively. Although solid CD instrumentation has been put into practical use, the preparation of samples with controlled parameters is seldom an easy matter. That is, it is quite difficult to control all the samples for quantitative comparison, for instance, a uniform thickness of film samples, membranes, and coated/deposited layers and the regular size of particulate hybrids. In addition, the low transparence of the samples may also give rise to difficulty in precise characterization. In fact, at present nearly all the characterizations are qualitative or semi‐quantitative for chiral solid products. With the development of academic instruments, this issue may be satisfyingly solved in the near future.
2) Enantiomeric excess values. For a most part of chiral applications ranging from asymmetric catalysis, chiral separation, to enantioselective detection, e.e. values provide more useful information regarding the chiral products derived from asymmetric catalysis reactions and the analytes of interest in chiral separation and enantioselective detection experiments. Unfortunately, in a majority of such experimentations, only few determined e.e. values. This situation may be associated with the difficulty in determining e.e. because of the challenges existing with instrumentation, as just mentioned above. Diverse instrumentations can be combined to measure e.e., for example, HPLC, CD, and optical rotation measurements may in combination assist in determining e.e. values. Moreover, the e.e. values reported in literature are not high enough. It indicates that there are still a large room for further improving the properties and functions of the chiral materials.
3) Synergistic effects. To solve the issue just aforementioned in the above section, making a full use and further improvement of the synergistic effects between graphene and chiral components may be an effective way. For example, in our chiral adsorption tests, we found that graphene can contribute to enhancing the total adsorption capacity, but meanwhile it decreases the enantioselective during adsorption. This is easy to understand, since graphene sheets have large specific surface area and the architectures derived from graphene sheets are frequently porous. Accordingly, how to rationally take advantage of the individual merits of the major building blocks forming the chiral hybrid materials and simultaneously circumvent the limitations of each one still remains as a challenge. Further optimization of the composition, micro‐ and macrostructure and morphology of the chiral hybrids may be helpful to overcome the problems.

### 5.2 Future Perspectives

In spite of the existence of difficulties and challenges, rational combination of chirality with graphene family materials has become one of the most active research subjects. The studies along this direction have demonstrated widespread impacts on multitude areas including material, chemistry, pharmaceutical, agrochemical, biomedical, etc. Especially, the potential chiral materials of the kind and their applications seem to be unlimited. Nonetheless, some issues related to the development and exploration of chiral hybrids derived from graphene also deserve much more efforts. Future research directions, perspectives, and in particular the issues requiring more consideration may include:

1) Constructional chirality and chiral metamaterials. Studies have demonstrated that graphene and the derivatives can be afforded with chirality through physical force driving the graphene sheets to twist and rumple in a single sheet or to stack in twisting.^[^ ^286^, ^287^, ^288^ ^]^ In other words, external chiral spices are no longer necessarily required. The as‐induced chirality, i.e., constructional chirality, has not been explored intensively yet. This type of chiral graphene materials is worthy to be investigated in depth, since they may show discernable properties and functions that cannot be observed in the analogues whose chirality is originated in additional chiral components. Additionally, GO sheets can form chiral liquid crystals and then assembled into macroscopic fibers with chirality.^[^ ^308^ ^]^ Graphene and its derivatives can possess luminescent behaviors by different manners (such as quantum confinement, nanographenes, and chemical functionalization).^[^ ^309^, ^310^ ^]^ The luminescence property may be combined with chirality to further develop circularly polarized luminescence materials, another category of quickly developing materials.^[^ ^311^, ^312^ ^]^ The potential applications of such a type of chiral graphene are still yet to be investigated. Graphene has been taken to develop metamaterials with distinguished electronic, optical, and magnetic properties. Their use potentials in solar‐thermal energy conversion, wave absorption, and electromagnetic shielding also cause a lot of interest.^[^ ^313^, ^314^, ^315^ ^]^ It is thus indicative that if graphene‐based metamaterials are combined with chirality, unprecedented materials with new phenomena and new utility may be highly anticipated. Graphene coupled with chirality provides huge opportunities for developing the newly emerging chiral metamaterials. For example, Masyukov et al. prepared terahertz chiral metamaterials with polarization manipulation ability, which is constructed by multilayered graphene.^[^ ^316^ ^]^ Hu et al. theoretically demonstrated that CD effects can be enhanced in chiral hybrid metamaterials composed of graphene and chiral metal nanocrystals at near‐infrared region.^[^ ^317^ ^]^ These studies indicate the bright prospects of chiral metamaterials developed from graphene.
2) Nano‐/microscaled chiral materials. Graphene and the analogs have been fabricated into various chiral composites, and in turn assembled into hierarchical architectures. Graphene can be electrospun into nanofibers.^[^ ^318^, ^319^, ^320^ ^]^ On the other hand, chiral membranes have been manufactured via electrospinning process.^[^ ^321^, ^322^ ^]^ It is hence hypothesized that chiral membranes based on graphene nanofibers may combine the merits of fibrous membranes and chirality. The as‐obtained chiral graphene derived nanofibrous membranes are expected to possess possible uses in chiral separation, chiral adsorption, chiral drug delivery, etc. In addition, nano‐ and microscaled chiral particles have been prepared using graphene nano‐ and microsheets.^[^ ^178^, ^180^, ^182^ ^]^ The chiral hybrid materials show enantioselectivity in chiral separation processes, e.g., in inducing enantioselective crystallization. Considering the advantages of nano‐ and microscaled architectures, graphene based chiral nano‐ and micromaterials are interesting both in nanofibrous and in particulate morphology.
3) Theoretical simulation. Experimental studies have made huge progress while theoretical simulations have been left far behind. Theoretical simulations may provide some guidelines for designing and preparing new chiral derivatives from graphene. For instance, molecular simulation has proved to be an effective tool to study graphene‐based membranes.^[^ ^323^ ^]^ Through comparing simulation predictions and the corresponding experimental measurements, insights into further improvement of membrane properties were acquired.^[^ ^324^ ^]^ Molecular dynamics simulations, together with experimental studies, help researchers to well understand the support effects of graphene in the supported catalysts thereof.^[^ ^325^ ^]^ Schwerdtfeger et al. proposed a new concept for functionalization of graphene aimed at chiral separation.^[^ ^326^ ^]^ Computational simulations show that attaching suitable chiral moieties to the pore rim in graphene prevents the passage of the distomer, while letting the eutomer pass. Based on simulation results, the introduced chiral molecules play the role of a “gatekeeper.” Nonetheless, theoretical simulations are still seldom used. They sometimes provide useful information that experimental studies cannot provide. Moreover, they may offer some important guidelines for design and preparation of chiral hybrids starting from graphene family materials.
4) Biological safety. One of the research directions concerning chiral graphene is to develop chiral composites intended for biological applications. Regarding the biological safety of graphene‐family materials, it is still under disputation.^[^ ^327^ ^]^ Some studies show that graphene and the derivatives are toxic to living organisms.^[^ ^328^, ^329^, ^330^ ^]^ Here are some typical reports. Graphene exerts toxic effects on bacteria,^[^ ^328^ ^]^ mammalian fibroblasts;^[^ ^329^ ^]^ graphene oxide results in severe and persistent lung injuries.^[^ ^330^ ^]^ Consequently, graphene family may pose potential risks for human health and even for other biological organisms. To elucidate the issue, more studies are still required; in particular, more in vivo studies should be accomplished for the chiral graphene materials targeted for biological uses.
5) Environmental concerns. Graphene‐related researches have grown at a spectacular pace in a wide range of disciplines. This situation has caused concerns about its health and ecosystem risks. Just like other materials derived from graphene, developing chiral graphene materials may lead to environmental pollution in three aspects presented below. The first one is associated with graphene. As typical 2D and advanced nanomaterials, the amount of graphene analogs increases rapidly each year, involving a large variety of scientific, engineering, technology, and material fields. The graphene family materials inevitably accumulate in environment and pose negative impacts on the environment and ecology systems.^[^ ^331^, ^332^, ^333^ ^]^ The second one is related to colloidal substances. It has been shown that polymeric, metallic, inorganic, and complex nanoparticles have aggregated in atmospheric air and natural waters, resulting in the emergence of new contaminants and the associated adverse effects.^[^ ^334^, ^335^, ^336^ ^]^ As far as graphene is concerned, micro‐ and nanoscaled graphene may be gathered in nature, causing drastic impacts on ecology. This issue may be explored together with the first one to exactly make clear the effects of graphene family materials (dimensions ranging from nano‐, micro‐ to macroscales) on nature environment, including both human beings and other organisms. The third one is connected with chirality. Owing to the enantioselective effects of chiral species, the design, preparation, and use of chiral matters, especially chiral drugs, agrochemicals, food additives, and fragrances, all highlight one in a pair of chiral enantiomers. If this condition goes on, it will unavoidably lead to chiral species accumulation in nature;^[^ ^337^, ^338^ ^]^ and eventually the original chiral equilibrium will be most likely destroyed. If so, catastrophic effects will occur to human beings and even the chiral nature. Accordingly, special attention should be paid to chiral graphene materials, from the beginning point of design, preparation, to use of them, and even the stage after use.

## 6 Conclusion

Graphene has become one of the star materials due to its fascinating structure, properties, and functions. Meanwhile, chirality is the fundamental attribute of nature. A judicious combination of them, i.e., developing chiral graphene materials, has proved to be a valuable research subject. The efforts have led to innumerable new functional materials integrating the advantages of graphene and chiral materials. The as‐created chiral hybrid materials have demonstrated promising applications in nearly all the specific areas associated with chirality, including asymmetric catalysis, enantiodifferentiating detection, and enantioselective crystallization. Chiralized graphene is also developed for biological applications. More recently, chiral luminescent materials were developed based on graphene. Considering the ever‐growing importance of biomimetic materials, layered‐structure graphene sheets and chiral helical macromolecules (both natural biomacromolecules and artificial helical polymers^[^ ^339^ ^]^) may be exquisitely designed and constructed to acquire advanced functionals materials like natural shells produced by biological organisms. The relevant studies clearly demonstrate the far‐reaching effects of chiralized graphene materials. This tendency will retain at least in the near future. The present review paper summarizes the explorations dealing with chiral graphene materials. The chiral species for chiralization of graphene family materials and their applications in the areas associated with chirality are systemically analyzed. Other relevant issues are also presented, including the newly emerging chiral graphene materials, the existing challenges, and the future perspectives. The continuing studies may not only lead to a great number of new chiral hybrid materials and accomplishment of practical applications, but also provide new insights into the homochirality of nature.

## Conflict of Interest

The authors declare no conflict of interest.
